# How many people die by suicide each year? Not 727,000: a systematic review and meta-analysis of suicide underreporting across 71 countries over 122 years

**DOI:** 10.3389/fpsyt.2025.1609580

**Published:** 2025-08-12

**Authors:** Nicola Meda, Ludovica Angelozzi, Matteo Poletto, Angelo Patane’, Josephine Zammarrelli, Irene Slongo, Fabio Sambataro, Diego De Leo

**Affiliations:** ^1^ Department of Neuroscience, University of Padova, Padova, Italy; ^2^ De Leo Fund, Padova, Italy; ^3^ Independent Researcher, Padova, Italy; ^4^ Padova Neuroscience Center, University of Padova, Padova, Italy; ^5^ Azienda Ospedale-Università Padova, Padova, Italy; ^6^ Italian Psychogeriatric Association, Padova, Italy; ^7^ Australian Institute for Suicide Research and Prevention, Griffith University, Brisbane, QLD, Australia; ^8^ Slovene Centre for Suicide Research, Primorska University, Koper, Slovenia

**Keywords:** misclassification, under-reporting, suicide, global burden, global health estimates

## Abstract

**Background:**

Suicide underreporting undermines accurate public health assessments and resource allocation for suicide prevention. This study aims at synthesizing evidence on suicide underreporting and to estimate a global underreporting rate.

**Methods:**

We conducted a PRISMA-compliant systematic review on suicide underreporting, following a pre-registered protocol. A meta-analytical synthesis was also conducted. Quantitative data from individual studies was extracted to provide an overall global estimate of suicide underreporting (42 studies covering 71 countries out of the initial 770 unique studies, spanning 1900–2021). Most studies used retrospective institutional datasets to estimate underreporting through reclassification of undetermined deaths or comparisons across databases. Demographic and geographic disparities were also examined.

**Results:**

The 42 studies selected provided some quantitative data on suicide underreporting for general or specific populations. 14 of these studies provided data to be meta-analyzed. The global suicide underreporting rate was estimated to be 17.9% (95% CI: 10.9–28.1%) with large differences between countries with high and low/very low data quality. In this scenario, the last WHO estimates of suicide deaths – corrected for underreporting – would be more than one million (1,000,638; 95% CI: 859,511–1,293,006) and not 727,000 suicides per year. Underreporting was higher in low- and middle-income countries (LMICs) with incomplete death registration systems, such as India and China (34.9%; 95% CI 20.3–53%), while high-income countries exhibited lower rates (11.5%; 95% CI 6.6–19.3%). Contributing factors included stigma, religiosity, limited forensic resources, and inconsistent use of International Classification of Diseases (ICD) codes. Gender and age disparities were notable; Female suicides and those among younger or older individuals were more likely to be misclassified.

**Discussion:**

Addressing suicide underreporting requires improving death registration systems globally, particularly in LMICs. Standardizing ICD usage, improving forensic capacity, and reducing stigma are critical steps to ensure accurate data. Heterogeneity, geographical disparities, temporal biases, and invariance of suicide underreporting for countries with low-quality data demand further corroboration of these findings.

**Systematic Review Registration:**

https://osf.io/9j8dg.

## Introduction

The World Health Organization (WHO) reported that approximately 727,000 deaths by suicide take place each year in the world (1, 2). However, this estimated toll relies on the quality of mortality data, which is poor for most countries in the world (3). The underreporting of suicide deaths has been a longstanding concern in epidemiology (4). Since Durkheim’s works, researchers and international organizations have sought to understand variations in national suicide rates, primarily relying on official statistics, which are acknowledged to be prone to underreporting and misclassification (5–7). A growing body of evidence highlights that suicides can be concealed within multiple cause-of-death categories, including injury deaths of undetermined intent (UnD) (5, 6), ill-defined and unknown causes, and accidental deaths such as accidental poisoning (7, 8). Theoretical frameworks suggest that misclassification could extend to almost all illness and injury categories (9), complicating the estimation of true suicide rates. Overall, it has been proposed that actual suicide rates may be underestimated by 10% to 30% (10, 11). However, figures can vary significantly according to the spatial-temporal coordinates and methodology of the studies, with a 1960’s study in Dublin, Ireland finding a 279% underreporting rate (12) and a 1988 study of Alaskan natives reporting 405% of underreporting (13), whereas more recent studies in westernized countries find approximately 8% (in Australia (14)), or even 1% (in Canada (15)). Nonetheless, any degree of underreporting would lessen the recognition of the magnitude of suicide and undermine the political will to develop an appropriate level of intervention (16, 17). The influence of social, cultural, and legal factors exacerbates underreporting. For example, stigma and the potential invalidation of life insurance claims discourage those who remain from correctly stating the manner of death (18). It has also been hypothesized that authorities are susceptible to ruling suicides as non-suicides, as a result of factors such as limitations in the system for collecting and compiling suicide data (e.g., data loss) (10, 19); factors pertaining to the knowledge or practices of individuals responsible for registration (20); cultural, religious, financial and legal considerations (e.g., precipitation of familial stigma, lower autopsy rates); and the ambiguity of some suicide deaths (21). These biases are further reflected in the variance of misclassification rates by the method of death, with suicides by hanging more likely to be accurately classified compared to those acted with less violent means (22, 23).

In a few studies, it has been found that some designated ‘accidental’ deaths could possibly contain underreported suicides, especially in the case of people aged 75 and over (24, 25), as 80% of fatal falls – often ruled as accidental deaths – are found in people of that age (26). Nonetheless, the category of deaths most responsible for suicide underreporting remains that of UnD (27–29). Demographic factors, including age, gender, ethnicity, and migration status, also influence the likelihood of misclassification, with suicides among younger individuals and internal migrants – moving from mainland to urban settings – found to be more frequently underestimated (20). Potential suicide misclassification is more likely among racial/ethnic minority due to limited suicide-specific information on these minority decedents (30–32). For example, suicide decedents without known mental health conditions have been reported to be more likely of racial/ethnic minority, relative to suicide decedents with documented mental health conditions (33). The upward correction of suicide rates for black decedents has been shown to reduce the gap in suicide rates between black and white adolescent decedents (18), but research remains limited on this topic.

At the national level, countries employ varying approaches to mitigate, or account for, suicide underreporting, ranging from comprehensive medico-legal investigations to combining suicide and UnD to estimate true suicide rates (34). Despite these efforts, the inherent ambiguity in categorizing UnD or accidental causes often perpetuates underestimation. For instance, the manner of some motor vehicle collision deaths, often presumed accidental, could conceal a suicide case (35–37).

The limitations of current methodologies necessitate several approaches to improve data accuracy. Enhancing the education and training of certifying and coding officials and systematically computerizing mortality data could also significantly improve the reliability of suicide statistics (32). To understand the prevalence of underreporting in suicide statistics, a few technical approaches have been developed and investigated to evaluate the magnitude of suicide misclassification. The first approach [e.g., (38)] uses detailed death records and assesses the process of classifying the cause of death by suicide. The second approach [e.g., (21, 39)] treats probable suicides (e.g., deaths by unknown causes or unidentified intent, and unintentional poisoning and drowning) as suicidal deaths and assesses whether alternative suicide rates including probable suicides are significantly higher than only those deaths which are classified as suicides in the original registry. The third approach [e.g., (31)] assesses whether suicides and probable suicides have similar background characteristics, such as gender, age, and medical history. The fourth approach [e.g., (40)] assesses whether suicide rates are correlated with mortality rates due to other causes, such as injuries of undetermined intent, unknown causes, or unintentional poisoning, under the assumption that suicidal cases are misclassified as deaths due to other causes, and thus, the underreporting of suicide results in the over-reporting of deaths by these causes. Moreover, Värnik et al. (28) proposed that suicide statistics can be considered valid if the mortality rate due to unidentified intent is below 2.0/100,000 and the proportion of deaths due to unidentified intent to suicide is below 20%.

Despite significant progress in understanding the drivers of suicide underreporting and how to mitigate it, systematic analyses of the reliability and validity of suicide data remain rare.

In this systematic review and meta-analysis, we synthesize the literature on suicide underreporting across 71 countries over 122 years to provide a worldwide perspective on suicide underreporting and thus estimate a more realistic number of suicides that take place each year in the world.

## Materials and methods

### Protocol and search strategy

This systematic review followed a pre-defined protocol available online (https://osf.io/9j8dg) and adhered to the procedures of the Preferred Reporting Items for Systematic Reviews and Meta-Analyses (PRISMA) statement.

Four investigators (NM, LA, MP, AP) independently and comprehensively searched PubMed, Scopus, and Web of Science databases with the following query string (applied to titles, abstracts, and keywords of the manuscripts): suicid* AND (underreport* OR misclassifi* OR “under reporting” OR “under-reporting”) from inception until 25 March 2024. A further manual search was conducted on the reference list of the included studies and relevant review articles. No *a priori* language restriction was applied. The investigators included any study for which a quantitative estimate of suicide under-reporting was computed, regardless of age, gender, ethnicity, health status, or nature of the data (aggregate or individual patient-level).

The reviewers independently established putative study eligibility through title/abstract reading, and when a consensus could not be achieved, a fifth reviewer (FS) was consulted. The full-text documents of potentially eligible articles were then retrieved, and the same investigators scrutinized each study for eligibility. The fifth reviewer was consulted if the authors could not reach a consensus for the definitive inclusion in the systematic review.

### Eligibility

Experimental, case-control, cross-sectional, or prospective studies were considered eligible. Commentaries, editorials, and reviews were excluded. A study was included if it provided at least one quantitative measure of suicide under-reporting (irrespective of comparator – i.e., another database, previous studies, vital statistics, etc. – to quantify the under-reporting) or presented data from which an estimate of under-reporting could be calculated.

### Data extraction

Duplicate records were excluded. For each eligible study we extracted the PMID/DOI, author, year, country/region, type of data (cross-sectional, prospective, etc.), years considered in the analysis, sample size, age of the sample, % females, if misclassification was the primary or secondary outcome of the study, which International Classification of Diseases (ICD) version was used for suicidal behavior definition, to which data authors’ suicide statistics were compared (e.g., unintentional deaths or other databases suicide statistics), % or another estimate of suicide under-reporting.

### Study quality

We rated the methodological quality of the studies using *ad hoc* criteria derived from the Newcastle Ottawa Scale. Specifically, we considered the database/baseline data to which suicide statistics were compared (e.g., comparing study original statistics to national vital statistics provides good quality data on national under-reporting), the sample size considered, the type of data (prospective > retrospective > cross-sectional), if misclassification was the primary outcome or not (see also Supplementary Materials).

### Data analysis

Whenever possible, we converted indices into under-reporting percentages. We excluded from the analysis all studies that reported correlation estimates or estimates for specific subpopulations [e.g., maternal mortality (41)], estimates referring to more than 30 years ago [e.g., (42)], or outlying estimates of underreporting (43, 44).

A random-effects meta-analysis was performed on logit-transformed underreporting proportions using the restricted maximum likelihood (REML) estimator, implemented via the *metafor* package (45) in R (a meta-analytical approach was not considered at the pre-registered protocol stage, but it was conducted after reviewers’ feedback). The logit transformation was chosen to stabilize variances and ensure the bounded nature of proportions was preserved. The primary pooled estimate was presented on the original scale using the inverse-logit transformation, along with corresponding 95% confidence intervals and a 95% prediction interval to reflect between-study heterogeneity. Heterogeneity was quantified using τ² (between-study variance), I² (proportion of total variability due to heterogeneity), Cochran’s Q statistic, and Akaike Information Criterion (AIC). To examine sources of heterogeneity, a mixed-effects meta-regression was conducted using WHO data quality tier as a categorical moderator. A separate meta-regression tested the NOS score as a continuous moderator to evaluate whether study quality influenced effect estimates. In addition, an inverse-risk weighted model was computed by weighting each study according to its NOS score, allowing higher-quality studies to contribute more heavily to the pooled estimate. The explanatory power of moderators was assessed using QM statistics and R², which reflects the proportion of between-study variance explained.

A leave-one-out analysis was conducted to evaluate the influence of each study on the pooled effect and heterogeneity metrics. Diagnostic statistics included studentized residuals, Cook’s distance, and changes in τ² and Q upon deletion of individual studies. Studies identified as highly influential were excluded in a follow-up sensitivity meta-analysis (reduced dataset, n = 11), and all models—including stratified, NOS-based, and inverse-weighted—were rerun on this reduced set. Forest plots were generated for each subgroup, and influence diagnostics were visualized to identify outliers and leverage points. The underreporting rate metrics derived from the meta-analysis were then used to infer a global underreporting figure.

In order to do so, we leveraged the data presented by the WHO on suicide worldwide in 2021 (2). Then, based on the meta-analytical findings, we considered that the average underreporting rates for each data quality score tier (WHO assigns countries to different categories according to the quality and completeness of data on suicide) could be inferred to the other countries belonging to the same tier. With that assumption, we computed a global estimate of underreporting.

## Results

### Sample characteristics

**Figure 1:**
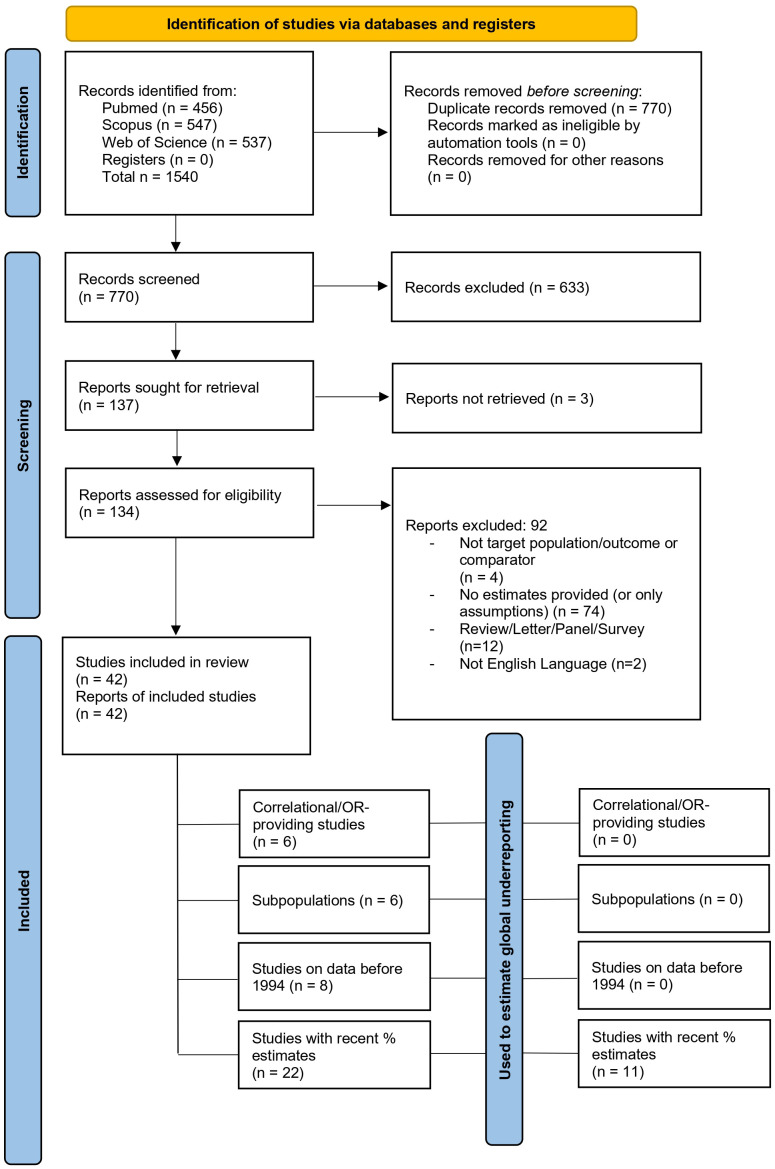
PRISMA flowchart. OR, odds ratio-providing studies.

**Figure 2:**
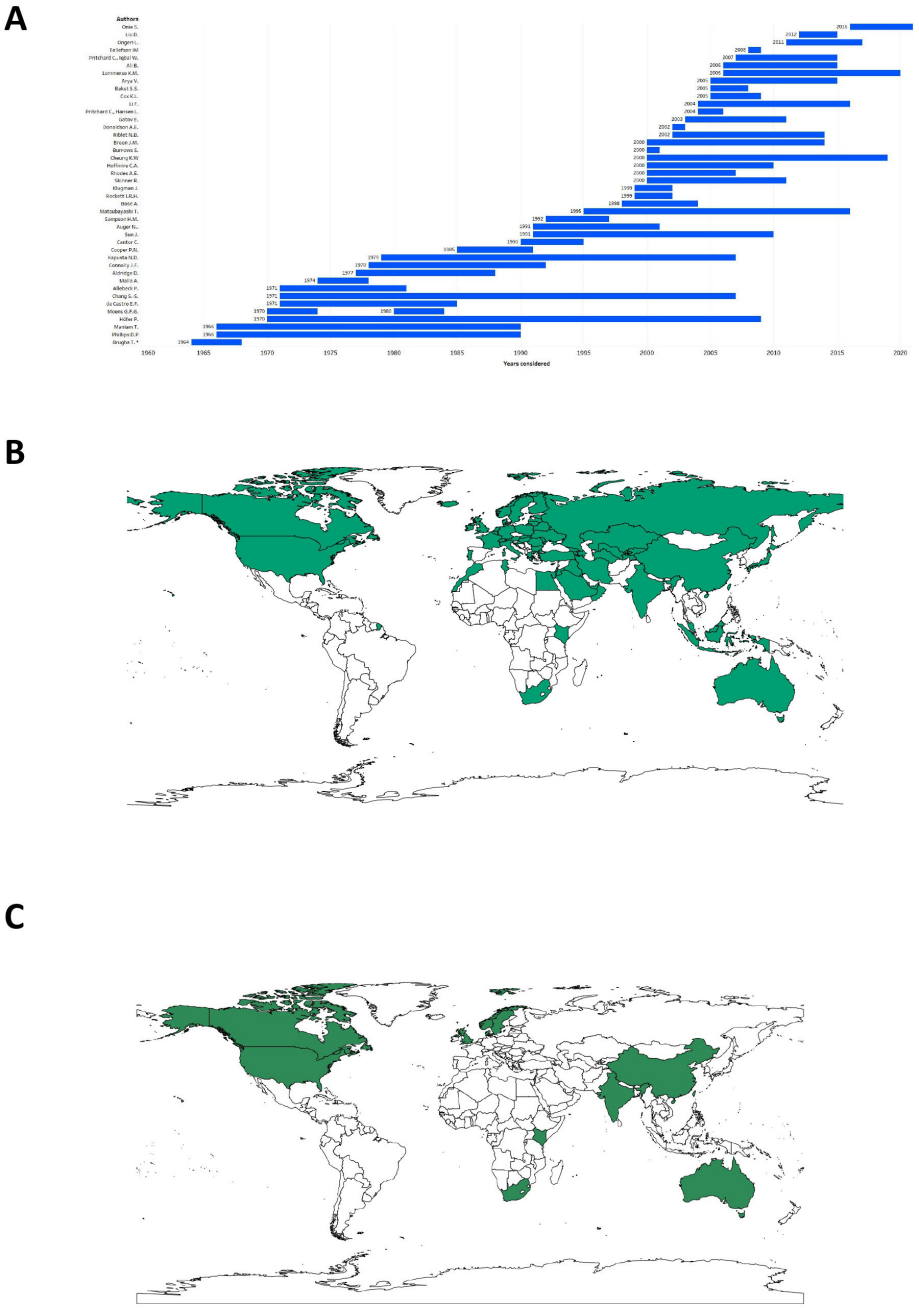
Countries or regions and year periods considered in the review. **(A)** Blue bars represent the years taken into consideration by each study to investigate suicide underreporting. For aiding visualization, the period 1900–1904 considered in the study by Brugha et al. was omitted; **(B)** Visualization of the geographical distribution of the seventy-one countries or regions (in bluish green) considered in this review; **(C)** Visualization of the geographical distribution of the 12 countries or regions (in green) considered in the meta-analysis.

The database search, after duplicate removal, brought 770 articles. After applying exclusion/inclusion criteria, the screening included 42 articles (**Figure 1**) that covered 71 countries (see also Supplementary Materials for study-country relationship). The included studies were published between 1978 and 2024, and all of them relied, at least partially, on data that institutions, organizations, governments, or consortia already collected. No study addressed suicide under-reporting with a prospective study design. The years considered by the single studies for establishing suicide under-reporting ranged from 1900 (46) to 2021 (44), while the length of the time periods being considered ranged from 1 single year (47–49) to 40 years (29) (**Figure 2A**). The majority of studies analyzed data from Western countries (**Figure 2B, C**). Five studies analyzed data from aggregated territories, such as Europe or European countries (48, 50, 51), Central Asia (51), Islamic countries (52), or Western Countries (17, 52). Five studies focused on single European countries such as The Netherlands (53), Norway (37), Poland (29), Portugal (40) and Sweden (54). Two studies leveraged data from Ireland (46, 55), two from the United Kingdom (56, 57), and one from Australia (58). Fifteen studies investigated data regarding North American territories, nine based on US data (32, 39, 49, 59–64) and six based on Canadian data (15, 21, 42, 65–67). Nine studies included data from Southeast Asia or neighboring territories (6, 16, 20, 41, 43, 44, 68–70). Lastly, two studies analyzed data from African countries (47, 71) and one from Israel (72). The data on the study characteristics are summarized in **Table 1** and are described in the next paragraph.

**Table 1:** Studies characteristics.

Authors	Year	Main data	Region	Years	Sample size (or suicide sample size)	Misclassification outcome	ICD version	Comparator	Age (suicide)	% Female (suicide)
Kapusta	2011	WHO	Europe/Asia	1979-2007	35 count.	Primary	ICD-9/ICD-10	UnD; Ill-defined; UnK	N.R.	N.R.
Cheung	2023	Clinical Data Analysis and Reporting System	Hong Kong	2000-2019	173 (15 suicides) + 5 Acc	Primary	ICD-MM	Any cause/manner of death except Acc	29 to 36	100%
Rhodes	2012	Ontario Coroners’ Reports	Canada, Ontario	2000-2007	Suicides 1,294; UnD 254; Acc 961	Primary	N.R.	Criteria for reclassification	10 to 25	25%
Chang	2010	EHR Department of Health	Taiwan	1971-2007	Suicides 76,015; UnD 8044; Acc 88,470	Primary	ICD-8/ICD-9	UnD/Acc paired for means of death	15+	33%
Bakst	2016	Israeli Central Bureau of Statistics	Israel, Tel Aviv	2005-2008	Suicide Probable 75, Suicide Verdicts 180, UnD 22	Primary	ICD-9	Unallocated Deaths	15+	Suic. Probable 32.0%, Suic. Verdict 23.0%
Li	2018	Shangai Police Records	Shanghai - Pudong	2004-2016	1,318 UnD of which 560 probable suicides	Primary	N.R.	UnD	15+	N.R.
Matsubayashi	2022	Vital Statistics of Japan	Japan, 47 prefectures	1995-2016	Suicides 604,726; UnD 43,644; UnK 127,039	Primary	ICD-10	UnD, UnK	20+	29.4% - 34.9% - 31.9% (suicides - UnD - Unk)
Pritchard	2015	WHO	20 Western Count.	2004-2006	20 countries	Primary	ICD-10	UnD/Acc	15+	N.R.
Breen	2018	Autopsy Records	Norway	2000-2014	338 MVC deaths	Secondary	N.R.	Natural and Acc death	18 to 64	0% - 28.6% - 18.9% (natural - nonculpable accident - suicides)
Ali	2022	NVDRS	USA (not all states)	2006-2015	Suicides 6,366; UnD 672	Primary	N.R.	UnD	10 to 19	23% - 28.6% (suicides - UnD)
Sun	2013	Shandong DSP system	China, Shangdong	1991-2010	16,028	Secondary	ICD-10	None - internal control	15+	43.1 - 50%
Onie	2024	National Indonesian Police Records, WHO GHE	Indonesia	2016-2021	1 country	Primary	ICD-10	Suicides	12+	41% (SRS Database)
Rockett	2006	US CDC	USA	1999-2002	N.R.	Primary	ICD-10	UnD, Acc	15+	N.R.
Sampson	1999	HM Coroner for South Yorkshire	UK	1992-1997	295: 233 suicide verdict + 62 open verdict	Primary	ICD-9	Criteria for reclassification	<45 and >45	N.R.
Connolly	1995	Central Statistics Office	Ireland, County Mayo	1978–1992	Suicides 143, UnD 41, Acc 15	Primary	N.R.	UnD, Acc	N.R.	N.R.
Burrows	2007	NIMSS *vs* medico-legal classification	South Africa	2000	Suicides 148, UnD 147	Primary	N.R.	UnD	15+	23% (in suicides with fully available data)
Riblet	2019	Veterans Affairs (SDR) + (RCA) database	USA	2002-2014	222 suicide (SDR) + 95 suicide RCA	Primary	ICD-10	Suicides	N.R.	N.R.
Lommerse	2024	ACMMM linked to database of CBS	Netherlands	2006-2020	68 suicides ante/postpartum	Secondary	ICD-10	Suicides	25-45	100%
Bose	2006	Christian Medical College, Vellore	South India	1998-2004	108,000 (total population)	Secondary	N.R.	Official data	0-80+	40%
Klugman	2013	Multiple Cause of Death individual-level files	USA	1999-2002	2,788 counties	Primary	N.R.	Acc, UnK, Natural, Homicides	15+	15% (mean value)
Allebeck	1986	Register of Stockholm County	Sweden	1971-1981	33	Secondary	ICD	None - internal control	20+	45%
de Castro	1989	Portugal Survey Data on CC	Portugal	1971-1985	1 country	Primary	ICD-9	Controversial cases	15+	23% - 28%
Phillips	1993	California Death Certificates	USA	1966-1990	7,811	Primary	ICD-7, ICD-8, ICD-9	UnK and Ill-defined, Acc	30,40,50, 60,70,80	31.5%
Cox	2017	US Army Criminal Investigation Cases Closed	USA	2005-2009	998 of which 510 suicides	Primary	N.R.	Acc; Homicide; UnD	N.R. (expect 18+)	N.R.
Liu	2020	Utah’s NVDRS	USA	2012-2015	2,665 overdoses (465 suicides)	Primary	N.R.	Reclassification of UnD overdoses	N.R. (expect 10+)	N.R.
Tøllefsen	2015	Death registers	Norway, Sweden, Denmark	2008	Suicides 600, Acc/UnD 600, Natural 600	Primary	ICD-10	UnD/Acc/Natural Deaths paired for means of death	18+	32%
Auger	2015	Canadian Census Mortality Follow-up study	Canada	1991-2001	Suicides 3,393, UnD 702	Primary	ICD-10	UnD	25+	40.2%
Donaldson	2006	Death Certificates Utah NVISS	USA (not all states)	2002	Suicides 87, UnD 84, Acc 41	Primary	ICD-10	UnD/Acc poisoning	18+	52.8%
Moens	1988	WHO	21 EU count.	1970-74, 1980-1984	21 countries	Secondary	ICD-8/ICD-9	UnD/Acc poisoning	5 to 24	31.5%
Arya	2020	NCRB, GBD	India	2005-2015	2,188,413	Primary	N.R.	GBD suicide estimates	15+	43% (GBD)
Cantor	2001	QSR, ABS	Australia	1990-1995	2,585 suicides (ABS); 2728 (QRS)	Primary	ICD-9	Suicide	1+	19.7%
Hoffmire	2020	State Mortality Project *vs* VA	USA, 23 states	2000-2010	32,516	Primary	ICD-10	Suicide	17+	2.7%
Cooper	1995	Data of HM Coroner for South Yorkshire (West)	UK	1985-1991	536 suicides: 323 verdict + 213 open	Primary	ICD, version NR	Suicide	N.R. (expect 0+)	40.2%
Skinner	2016	Statistics of Canada mortality	Canada	2000-2011	11,149	Primary	ICD-10	All death manners by poisoning	15+	N.R.
Gatov	2018	OCC linked to ORG-D, Statistics Canada’s Vital Statistics database	Canada, Ontario	2003-2012	11,697	Primary	ICD-9, ICD-10	Suicides, UnD/Acc deaths	21+	24.9%
Malla	1983	Registrar of Births and Deaths	Canada, N&L	1974-1978	Suicides 103, “unexplained” 104	Primary	N.R.	Suicides, UnD/Acc, Uninvestigated deaths	N.R.	N.R.
Aldridge	1991	Death records from eight hospitals	Canada, N&L	1977-1988	63 suicides	Primary	N.R.	Suicides, UnD/Acc	10 to 19	16.7%
Maniam	1995	Department of Statistics	Malaysia	1966-1990	1 country	Primary	ICD-7, ICD-8, ICD-9	Suicides, UnD violent deaths	0+	33%
Höfer	2012	Polish Central Statistics Office	Poland	1970-2009	190,559 suicide	Primary	ICD-8, ICD-9, ICD-10	Suicides, UnD, UnK, Acc	0+	12%
Ongeri	2022	Verbal autopsies Data from the (HDSS)	Kenya, Kisumu County	2011-2017	7,915	Primary	ICD-10	Suicides, Acc	17 to 73	N.R.
Pritchard	2020	WHO	Islamic and West count.	2007-2015	25 countries	Primary	ICD-10	Suicides, UnD/Acc	N.R.	N.R.
Brugha	1978	Coroners’ inquest records	Ireland (Dublin)	1900-1904	1,412 inquest records (1900–1904)	Primary	N.R.	Reclassification, UnD/Acc deaths	N.R. (major clusters 25-34)	24.1%

ACMMM, Audit Committee Maternal Mortality and Morbidity; Acc, Accident/Accidental; CBS, Central Bureau voor de Statistiek (The Netherlands); CDC, Centers for Disease Control and Prevention; Count., countries; DSP, Disease Surveillance Point; EU, European/Europe; HER, Electronic Health Records; GBD, Global Burden of Disease; GHE, Global Health Estimates; HDSS, Health and Demographic Surveillance System; ICD, International Classification of Diseases; ME, Medical Examiner; MVC, Motor Vehicle Collision; NCRB, National Crime Records Bureau (India); NIMSS, National Injury Mortality Surveillance System (South Africa); NVDRS, National Violent Death Reporting System (USA; South Africa); NVISS, National Violent Injury Statistics System (USA; Utah); N&L, Newfoundland and Labrador (Canada); OCC, Office of the Chief Coroner (Canada); ORG-D, Office of the Registrar General–Deaths (Canada); QSR, Queensland Suicide Register (Australia); RCA, Root-Cause Analysis (USA); SDR, Suicide Data Repository (USA); SRS, Sample Registration System (Indonesia); UnD, Deaths of undetermined intent; UnK, Deaths of unknown cause; VA, Veteran Affairs (USA); WHO, World Health Organization.

Suicide under-reporting was the primary outcome in thirty-six studies; only six studies evidenced suicide under-reporting coincidentally or investigated it as a secondary outcome (37, 50, 53, 54, 68, 69). To provide an estimate of suicide underreporting, most studies compared reported suicide rates to more than one rate among the following: UnD, unknown cause/manner of death, ill-defined codes, accidental deaths, any deaths, self-defined criteria for establishing the manner of death or internal controls (e.g., previous data on underreporting in the same population). Some studies, instead, employed one single comparator: suicide (57) as a manner of death (e.g., examining putative deaths by suicide through self-defined criteria); reclassifying UnD or comparing suicide rates to UnD (20, 21, 32, 39, 47); recoding controversial cases (40, 56, 65); using internal controls (54, 68); accidental deaths (37); unallocated causes of death (72); official/governmental data or other established databases (44, 53, 58, 60, 64, 69, 70). Globally, UnD, of which the cause was unknown or unallocated causes of death were used to some extent in 25 studies. Accidental deaths were used as a comparator in 18 studies. Comparison of databases, a re-examination of suicide deaths, manner/causes of death recording, or internal controls were employed in 23 studies. Any cause or manner of death, death by natural causes, by homicide, or recoding ill-defined codes were methods of comparison in 8 studies. However, 14 studies did not reference using the ICD (any version) to provide unequivocal codes (20, 32, 37, 39, 42, 46, 47, 55, 61, 63, 65, 67, 69, 70). To describe the sample size considered in this systematic review, we divided the studies into those for which: i) the sample size being considered is the number of countries or counties (i.e., the study analyzed national or regional-level suicide trends); ii) the sample size referred to UnD or a pool of deaths, where suicide is *a priori* considered to be more likely to be misclassified; or iii) the sample size being considered is the number of suicides. In the first case, 104 countries (some over-sampled, mainly European countries) were considered by studies covering country-level aggregated data and 2,788 counties by a single study (61). Another community-based study focused on 108,000 people (69); in the second case (ii), 7,374 people comprised the sample. In the latter case (iii), a total of 3,155,953 “reported” suicide cases were identified. For one study (55), the sample size was not explicitly stated (only suicide rates were available). Regarding the age ranges considered, most of the studies (n=11) considered people aged 15+ or all ages (n=5). In a significant number of studies (n=9), the age of the population was not explicitly stated or reported. In 9 studies, specific age ranges were considered (e.g., 17-73; 5-24). In the remaining studies (n=8), population age was heterogeneous with an age lower limit varying from 12 to 21 and no upper age limit. Regarding the gender distribution in suicide across the studies, 12 studies did not state it. Apart from two studies that investigated suicide during peri-partum (41, 53), the percentage of females reported by the studies ranged from 15% (61) to 52.8% (49). Data on the demographics of the target population of each study is included in **Table 1**.

### Underreporting estimates

To describe the underreporting estimates, the studies included in this review are presented according to this stratification: i) geographic zone; ii) target population; and iii) type of estimate (percentage of underreporting, correlation between rates, or other).

### Aggregated territories

Five studies took into consideration aggregated territories comprising more than one country. The study by Moens et al. (50) took into consideration WHO mortality rates between 1970–1974 and 1980–1984 in 21 European countries. For all ages being considered in the study - which particularly stressed the phenomenon of suicide in adolescents/young adults - the lowest rates of suicide under-reporting computed by the authors ranged from 13% (in males) to 20% (in females) between 1980-1984. However, such percentages are certainly an overestimation of under-reporting as the authors assume that UnD and accidental poisoning deaths are all suicides. Tøllefsen and colleagues focused on the suicide death registers of Norway, Sweden, and Denmark (48). The authors took into consideration only 2008 as the year for their analysis. Still, they paired a total of 1,800 accidental, UnD, natural, and suicide deaths of people aged 18 or older by methods of death (e.g., poisoning) to control for possible bias of the method in misclassification. The authors asked forensic pathologists, psychiatrists, and an expert coder to re-classify the deaths. The agreement between official suicide statistics and expert opinion ranged between 81% (Swedish dataset) and 90% (Danish dataset). The primary source of disagreement was the reclassification, by experts, of suicide deaths as UnD. However, taking into account other sources of suicide misclassification, the authors concluded that any change in suicide rate would be small and would not significantly impact suicide statistics. One pivotal point is that expert coder’s and psychiatrist’s opinions diverged significantly concerning the reclassification of suicide deaths as UnD, underscoring the role of sufficient information to reach a verdict and that the UnD codes, more than accidents, can hide deaths by suicide.

In a study that considered 35 European and Central Asian countries across 29 years (from data based on the WHO “European Health for All Database), Kapusta et al. (51) estimated that each 1% increase in autopsy rate is associated with an increase of 0.38 - 0.48 suicide per 100,000 population, after controlling for proper confounding variables and other possible sources of misclassification. Although it is difficult to infer how many deaths by suicide were misclassified during the study period, this data supports the crucial role that “sufficient” information (e.g., those derived from autopsy) can provide to coders and experts in ascertaining the intent to die of the decedent (8).

The study by Pritchard and collaborators (17) analyzed WHO Annual Mortality Statistics of suicide, accidental and UnD death rates across twenty Western countries between 2004 and 2006. The authors identified a significant and strong correlation between suicide rates and rates of death by suicide. Moreover, for people aged 75 or over, the rates of UnD were significantly higher in predominantly Catholic countries, suggesting a putative role of religiosity as a moderator of suicide death misclassification. This article highlights the strong likelihood of suicide under-reporting across several age ranges, in particular in people of older age, and how increasing rates of UnD deaths with age might be inflated by (purposefully or not) suicide misclassification. Lastly, Pritchard and colleagues further examined the role of religiosity in suicide under-reporting by comparing four European countries deemed strongly orthodox/catholic to 21 predominantly Islamic countries (52). WHO mortality data updated to 2018 were leveraged to compare suicide rates to UnD + accidental death rates, with the assumption that a ratio of UnD + accidental rates to suicide rates greater than 2 likely indicates some degree of suicide under-reporting. 19 out of 21 predominantly Islamic countries had ratios greater than 2, with almost half of them having a ratio of 15 or higher. However, in 16 of these countries, the number of reported suicides per 100,000 people is below 3, which is approximately 1/3 of the global average of suicide rates, according to the WHO 2019 report (1).

### Western countries (i): Europe and Australia

This section includes seven studies that leveraged data from Norway, Sweden, Portugal, Poland, The Netherlands, and Ireland (2 studies – Dublin and County Mayo), two studies on UK (South Yorkshire) data, and one on Australian data. All of the above studies, but one (40), report a definite sample size with an estimate of under-reporting. De Castro (40) did not report the number of suicides in Portugal in the period of interest (1971–1985) but showed an interesting association between the adoption of ICD-9 in 1979 (which included “controversial cases” – CC – category) and a subsequent increase in controversial cases of 1200 – 2100%. Given that the socio-demographic profile of controversial cases is similar to that of suicide, the authors speculated that the adoption of the CC category implied a suicide under-reporting of 30%.

Breen et al. (37) investigated the percentage of suicides in 338 motor vehicle collisions (MVCs) with culpable drivers in Norwegian forensic autopsy records. Although the nature of the data did not allow for a direct estimate of suicide under-reporting, the authors found that 10.9% of MVCs were deemed to be suicides, thus pinpointing the importance of investigating MVCs, especially single-driver ones (73, 74), as possible suicides. A Swedish study on suicide and violent deaths in people with schizophrenia between 1971 and 1981 (54) concluded that out of 34 deaths ruled as accidental or UnD in the Death Register of Stockholm County, 7 were instead deaths by suicide. Of the 33 deaths ruled as suicide at first evaluation, none of them were re-classified to other manners of death, meaning that the misclassification of suicide in this sample was 16.5%. Höfer and collaborators (29) conducted the study with the longest observation period in this review. The authors inspected data from the Polish Central Statistics Office on 190,559 deaths by suicide registered between 1970 and 2009. Based on the comparison with UnD, unknown cause, and accidental death rates, the authors evidenced that misclassification of suicide ranged from 27 to 30%, with gender differences suggesting a greater undercounting of female suicide (32-35%) than male suicide (25-29%). Brugha and Walsh in 1978 (46) analyzed 1,412 inquest records of the 1900–1904 period in Dublin to compare the degree of under-reporting in those five years to previous findings regarding 1964 and 1968. Based on coroners’ verdict of suicide, in the Dublin County Borough at that time, 29 people took their lives and 51 deaths remained undetermined. Brugha and Walsh identified 28 more deaths by suicide “*because the evidence in the record showed that the deceased understood the fatal consequences of his self-destructive action and intended that consequence*” (pg.178). Thus, the authors found that the under-reporting rate between 1901–1904 and 1964–1968 remained unvaried between 50 to 75%. In County Mayo (Ireland), Connolly and Cullen (55) examined the files of all deaths reported to the County coroners between 1978-1992. The authors were confident that they had “clear evidence” (from reports) that 220 deaths were by suicide, although 35% of them were either misclassified (to UnD or accident) or unregistered. In the UK, Cooper and Milroy (57) inspected the files of the HM Coroner for South Yorkshire (West) from 1985 to 1991. The authors pre-defined suicide according to the definition of Kennedy et al. (75), intent was inferred based on notes, *prima facie* evidence, or circumstantial evidence as suicide being the most reasonable manner of death. In this way, the authors identified 323 coroner’s suicide verdicts and 213 open verdicts, which were considered be suicide. Therefore, 39.7% of suicides were deemed to be under-reported. Sampson and Rutty analyzed suicide underreporting in the same area (South Yorkshire – West) between 1992 and 1995 (56). Data were collected from the same coroner’s office. The review of the verdicts followed overlapping criteria as those in the study by Cooper and Milroy (57). In this study of 295 suicides, of which only 233 received a suicide verdict, the authors evidenced a lower percentage of suicide underreporting with respect to Cooper and Milroy’s study, with an estimate of 21%.

The last study in this region was conducted in The Netherlands and investigated suicide under-reporting specifically for child-bearing women (aged 25-45) and 1 year postpartum (53). In this specific case, the goal of the study was to evidence how many suicides during pregnancy or postpartum were not reported to the Audit Committee (for) Maternal Mortality and Morbidity, assuming that all deaths by suicide were reported to the Central Bureau of Statistics (0% of underreporting). In this optimistic framework, the under-reporting to the Committee was 70.6%, meaning that only 20 deaths by suicide (out of 68) were known to have taken place during pregnancy or postpartum, thus hindering inquiries and care improvement.

In Australia, Cantor and collaborators (58) compared the Queensland Suicide Registry (QSR) to the Australian Bureau of Statistics (ABS) to evince the magnitude of under-reporting of suicide in the national database between 1990 and 1995. The QSR identified 2,728 deaths as suicides, whereas the ABS officially reported 2,585 suicides. Thus, the underreporting of suicide in Australia was estimated to be approximately 5.2%.

### Western countries (ii): USA and Canada

Most of the studies (n=15) were conducted in these regions: six in Canada (2 in Ontario and 2 in Newfoundland and Labrador) and nine in the US territories. All of these studies reported a sample size except for (59). In the latter study, Rockett and associates extracted data from the US Center for Disease Control and the Prevention’s Web-based Injury Statistics Query and Reporting System on suicides between 1999 and 2002 to provide a raw estimate of suicide underreporting according to “*race*” (59). To do so, the authors assumed that the “real” suicide rate would be the result of the officially reported suicide rate and UnD. Under this assumption, the authors reported that “real” suicide rates in black males, across all ages, would be 18.7% higher, while the suicide rates in black females would be 34.3% higher than officially reported.

The study by Phillips & Ruth (62) investigated suicide underreporting using data from 1966 to 1990 by evaluating the California Death Certificates. The authors took into consideration 7,811 deaths by suicide. They compared them to deaths by single-car accident, accidental poisoning, pedestrian accidents, and deaths by unknown or ill-defined causes for people aged 30, 40, 50, 60, 70, and 80 years. Between 1966 and 1990, suicide under-reporting rates ranged between 0.84% and 4.23% (percentage of suicide misallocated to pedestrian deaths, barbiturate deaths, and deaths from ill-defined causes). However, when considering “*race*”, whites have an estimated suicide underreporting of 3.3%, whereas blacks have 14.9%. Klugman and colleagues (61) investigated if and how suicide underreporting might be influenced by variations in the medico-legal system. The authors analyzed aggregated data for 2,788 counties in the United States between 1999 and 2002 and compared variations in suicide rates to variations in deaths by accident, homicide, illnesses, and UnD. The authors reported that counties with medical examiners (all of whom were appointed) had higher suicide rates for both female (5.26/100,000/year) and male (27.66/100,000/year) decedents than those counties with elected coroners (4.66 and 25.94/100,000/year for females and males, respectively), resulting in a percentage of suicide underreporting ranging between 6.2 and 11.4% according to gender (61). Hoffmire and colleagues (64) took into consideration the years between 2000 and 2010 to compare the State Mortality Project statistics and those of Veteran Affairs (VA). In their analyses covering 23 states in the US, the authors included 32,516 deaths by suicide. When the two systems of data collection were compared, the authors reported that the State Mortality Project suffered approximately a 24% underreporting of suicides when compared to VA/Department of Defense (DoD) data, mainly due - according to authors - to the complex validation processes to identify veteran suicide decedents. Also, Riblet et al. queried the VA/DoD suicide data to compare suicides detected through National Death Index (NDI) record linkage and Root-Cause Analysis (RCA) that happened 7 days after discharge from an inpatient mental health unit (60). Their study period covered 2002 and 2014 and evidenced that suicides in the NDI dataset were 222, whereas the RCA only reported 95 deaths by suicide. The authors stated that RCAs were conducted only a non-random subsample of the suicides in the week after discharge, thus implying a high suicide underreporting (64.8%) with respect to NDI, especially for deaths due to suicide from overdose. Conversely, NDI misclassified 13 deaths by suicide (according to RCA) to accidental poisoning or other causes (suicide under-reporting in NDI was 5.5%). In the context of misclassification in army suicides, a study by Cox et al. (63) took into consideration a 5-year period, from 2005 to 2009, to investigate misclassification of suicides (n=510) to homicide (n=14), accidental death (n=426), or UnD (n=48). Data on the demographics of the sample were not reported. Although the data were derived from criminal records, and thus obtained after thorough investigations to decide whether a crime occurred or not, the authors estimated a possible underreporting of 8.2%. It is noteworthy that 23 cases of suicides (4.5%) were not deemed to be “definitely” suicides, thus mitigating the absolute number of suicides being misclassified. Two studies queried Utah’s National Violent Injury Statistics System (NVISS). Donaldson and colleagues (49) focused their analysis on deaths by poisoning that happened in 2002 and compared suicide as a manner of death (n=87) to UnD or accidents (n=84 and n=41, respectively). Using Classification and Regression Tree (CART) analysis, the authors sought to reclassify UnD as either accidental or suicide deaths. Assuming that suicides and accidental deaths used to train the CART were “true” suicides/accidents, the algorithm suggested that the underreporting of suicides due to poisoning was 29.3%. This would result in a global underreporting of suicides (all causes) of approximately 10% (the authors reported that there were 342 suicides from all causes in Utah in 2002). Liu and colleagues (39) applied several machine-learning algorithms (e.g., neural networks, random forest) to reclassify deaths by overdose of undetermined intent (n=715) as either accidental (n=1,485) or suicide (n=465) as a manner of death. The period being considered was 2012-2015. Data on demographics were partially not reported. The authors reported that all algorithms had accuracy ranging from 92.3 to 94.6% and estimated a mean percentage of suicide underreporting of 33% (which increased from 29% in 2012 to 37% in 2015). The last study based on US data being considered was by Ali et al. (32) who examined the National Violent Death Reporting System (NVDRS) Restricted Access Database (RAD) files, a population-based surveillance system managed by the US CDC for deaths by suicide (n=6,366) and UnD (n=672) occurred between 2006 and 2015. The authors did not compute an overall suicide under-reporting percentage. Still, they evidenced that equivocal deaths of black people would be 40% more likely (all else being equal) to be classified as of undetermined intent than those of white people.

Six studies conducted in Canada were included in this systematic review. The less recent ones were by Aldridge and St. John (42) and Malla and Hoenig (67) and both focused on the regions of Newfoundland and Labrador. Malla & Hoenig focused on 1974–1978 to investigate if, among the 104 unexplained deaths in those years, there was evidence of suicide misclassification (cases reported as suicide were 103). By further investigating the equivocal deaths, through records of forensic pathologists, the authors conclude that 14 unexplained deaths were unequivocally deaths by suicide. Moreover, the authors showed that 58 of 104 unexplained deaths were not investigated. In the most conservative scenario (only 14 suicides misclassified), the percentage of underreporting would have been 11.9%; moreover, if all non-investigated deaths were to be considered suicides, the percentage of under-reporting would have been 41.1%. Aldridge and St. John focused instead on 63 deaths of adolescents (10 to 19) that happened between 1977 and 1988 and were recorded in one of the eight hospital pathology departments in the province and from the office of the Chief Forensic Pathologist. From the records they retrieved, the authors evidenced that only 36 of 63 deaths were designated as suicides on death certificates, thus producing an underreporting of 42.8%. Two studies focused on the region of Ontario. Rhodes et al. (65) queried the Ontario Coroner’s reports produced between 2000 and 2007 on 1,294 suicides, 961 accidental deaths, and 254 UnD of people aged 10-25. The authors produced two types of reclassification: 1) all UnD being reclassified as suicides; 2) reclassification of UnD/accident according to literature criteria of higher likelihood of death by suicide. The stricter criteria (2) indicate that 14.3% of accidental/UnD were more likely to be suicides (number of suicides reclassified=186). The study led by Gatov et al. (15) focused instead on 11,697 coroner-confirmed suicides of people older than 21 between 2003 and 2012, which were linked to Canada’s vital statistics. The concordance between the two datasets was very high, steadily increasing through the years being considered, with only 1.2% of deaths by suicide being misclassified in the vital statistics in 2012 (mean of 3.16%). Lastly, two more studies focused on Canadian data mortality. Auger et al. (21) leveraged the Canadian Census Mortality Follow-up study (1991–2001) which recorded 3,393 suicides and 702 UnD of people aged 25 or older. The nature of the dataset implied that an accurate estimate of suicide underreporting could not be computed. Thus, the authors summed the UnD to suicide cases to estimate that under-reporting might lower suicide rates by 10 to 38%. Lastly, Skinner et al. (66) studied the Canada mortality statistics from 2000 to 2011 for 15+ year-olds, which included 11,149 suicides, 14,317 accidental poisonings, and 5,248 undetermined poisonings. The authors evidenced an ever-increasing rate of poisoning of undetermined intent to UnD (47% to 80% in the study period), with the ratio of suicide through poisoning to suicide (all causes) showing no significant trends. This study, although it did not provide an estimate of underreporting, strongly indicated that poisoning suicides could be misclassified as poisoning of undetermined intent.

### Asian countries

Nine studies were conducted in Asian territories. Most studies were conducted in China or regions near China (n=4). A study was conducted in 47 prefectures in Japan. Four other studies were conducted in South-East Asia (South India, India, Indonesia and Malaysia). All studies except one reported a sample size (6). Maniam studied the publications of the Vital statistics of West Malaysia between 1966 and 1990 to evince how suicide rates were correlated with violent UnD (6). The author showed a high negative correlation between the two rates, especially marked after the introduction of the ICD-8 (in which a death could be classified as “other external causes [of death]/other violence”). An estimate of underreporting, based on the assumption that all “undetermined [intent] violent deaths” are indeed suicide, was also provided. In contrast to the official incidence of suicide in the 1980s (1.5/100,000/year), the author calculated a “true” incidence rate of 8-13/100,000/year (thus, considering the lower value, 81.2% of under-reporting). In a study based on a rural and peri-urban population in South India, Bose et al. (69) used surveillance data covering a population of 108 thousand people over 7 years (1998–2004). From their research, a suicide rate of 82.2/100,000/year was found. On the basis of other studies conducted in rural areas of India, either reviews (76) or studies examining the National Crime Records Bureau (NCRB) statistics (77, 78), suicide underreporting could widely range, from 24% to 77.3%.

Arya and collaborators (70) compared the NCRB to Global Burden of Disease (GBD) estimates to gain insights into the suicide underreporting of Indian Vital statistics. Their study covered the years from 2005 to 2015 and observed 2,188,413 suicides (according to the GBD). The authors evidenced that the Vital Statistics underreporting was approximately 37% (or 802,684 additional suicides found in the GBD estimates), with significant differences according to gender (male underreporting of 21-31% and female underreporting of 47-54%) and age (higher underreporting in the 15–29 and over 60 age ranges). Another study from Southeast Asia, by Onie et al. (44), evidenced the highest percentage of suicide under-reporting in contemporary times. The observation period was 2016-2021. In their study, the authors compared the National Indonesian Police Records (which are reported to be the official sources of suicide statistics in Indonesia) with four other data sources: death registry data (from the Ministry of Home Affairs of Indonesia), a provincial survey (Village Potential Survey), a sample registry system (developed by the National Research and Development Center covering almost 5 million people), and the WHO Global Health Observatory (GHO). Assuming 100% coverage and correct classification of suicide by the WHO GHO, suicide under-reporting by official statistics ranged between 86.2% and 90.4%, this means that for each suicide effectively reported, almost one other suicide was not reported or misclassified. Sun and colleagues studied suicides in Shandong, China, between 1991-2010 (68). The authors queried the mortality database of the Shandong Disease Surveillance Point, provided by the Shandong Provincial Center for Disease Control and Prevention, which collected data on 16,028 deaths by suicide. Given that misclassification was a secondary outcome for this study, no control group or specific analysis of this outcome was conducted. However, the authors reported that they found a consistently small percentage of “injuries with intention unspecified” in the dataset (<5% of all injury deaths) and stated that some deaths by suicide were likely to be underreported in this category. However, data on possible misclassification of deaths by suicide with less violent means to other categories was not reported. Li and coauthors (20) queried Shangai Police Records to reclassify UnD in the District of Pudong between 2004 and 2016. Among 1,318 UnD, 560 were classified as ‘probable’ suicides (“*probable suicides were suicides of high certainty, but still, this cannot be ascertained with certainty*”). In the same district, 2,407 ‘official’ deaths by suicide happened in the same period. Based on the data provided by the authors, suicide under-reporting in the district was approximately 18.8%. Chang and collaborators (16) analyzed 37 years of data provided by electronic health records on suicide in Taiwan. The authors divided the period into two timeframes due to the increase in the use of the UnD category in 1980. The authors found that between 1971–1989 there were 34,230 suicides, 433 UnD, and 44,146 accidental deaths. In 1990-2007, 41,785 suicides, 7,611 UnD, and 44,324 accidental deaths were observed. The authors compared the aggregated demographics of the decedents and rates of the three manners of deaths to conclude that, in Taiwan, in the period 1990-2017, suicide deaths were under-reported by at least 30%, with UnD, accidental poisoning by pesticide and accidental suffocation as the categories most likely to account for suicide misclassification. Lastly, Cheung et al. (41) studied maternal mortality statistics in Hong Kong between 2000 and 2019. The authors compared cases of death as reported by the Vital Statistics of Hong Kong with deaths registered in the hospital-based cohort. Although suicide misclassification was not the primary outcome of this study, the authors found that out of 173 maternal deaths (i.e., happened during pregnancy, childbirth, or less than a year from the end of the pregnancy), 74 happened during pregnancy or in the first 42 days after delivery, and suicide was the leading cause of death (n=15, 20.3% of deaths). In addition, none of the cases of suicide was reported by the vital statistics (underreporting 100%).

Matsubayashi and Ueda studied Japan Vital statistics for 47 Japanese prefectures from 1995 to 2016 (43). The authors investigated if 604,726 suicides, 43,644 UnD, and 127,039 deaths of unknown causes were to some degree inversely correlated to each other, and if major socioeconomic causes of suicide (e.g., unemployment) had any relationship with the deaths due to unknown intent or cause. Based on their correlational analysis, the authors found no evidence of a relationship between the three manners/circumstances of death. Moreover, the authors found no statistically significant relationship between these death rates and the socio-demographic indicators, except between the divorce rate and deaths due to unknown causes rate among men aged 40–64 years. In general, however, Matsubayashi and Ueda reported that there would be no strong evidence for the possibility of under-reporting suicidal deaths in the Japanese context.

### Middle-Eastern countries

Only one study from Israel could be included in this systematic review. Bakst et al. (72) queried the Israeli Central Bureau of Statistics and thoroughly investigated intentionality by retrieving supplementary information from Israel’s emergency medical services, hospital medical records, National Institute of Forensic Medicine reports, and criminal investigations division of the local (District of Tel Aviv) police departments. For the years 2005 and 2008, 180 officially defined suicides, and 2,964 other “unallocated” death cases were identified. Of these 2,964 deaths, 75 were deemed probable suicides (considered by the authors as definitive suicide cases, albeit not officially certified as such). Thus, suicide underreporting was 29.4%.

### African countries

Two studies were from African countries. Burrows and Laflamme (47) queried the National Injury Mortality Surveillance System (NIMSS) and compared its suicide classification to standard medico-legal investigations (which were considered the gold standard). A total of 148 deaths by suicide and 147 UnD that occurred in 2000 were retrieved from the datasets (yet, in the medico-legal system, one-third of cases could not be tracked, had not been finalized, or had unclear outcomes). However, for the data available, while considering UnD misclassification to suicide, the net percentage of underreporting was 11.5% (14 suicides misclassified as UnD – under-reporting – and eight UnD misclassified as suicides – over-reporting; thus, 115 NIMSS suicides were instead 107 suicides – stripped of over-reported cases – which become 121 suicides when considering underreporting cases). Ongeri and colleagues (71) compared Kisumu County, Kenya’s Health and Demographic Surveillance System (HDSS) data to verbal autopsies conducted by the authors. Data referred to 2011–2017 and 7,915 deaths in a population of 154,140 residents. After combining confirmed and suspected cases and adjusting for missing data, the authors, using verbal autopsies, found suicide rates approximately fourfold higher (14.6/100,000/year) than official statistics (3.6/100,000/year) – marking large suicide under-reporting.

### Global estimate of suicide underreporting

To produce a useful, global estimate of suicide under-reporting, and to increase generalizability, we opted to exclude from this pooled analysis: i) the studies that focused on specific subpopulations or (32, 39, 49, 59, 64, 65); ii) studies for which WHO GHO estimates were used to compute national suicide underreporting (44); iii) studies that provided correlational data (6, 17, 29, 51, 66), odds ratios for classification to undetermined or accidental category (52), vague estimates (37, 68), or estimated under-reporting in local or specific databases not related to national statistics or, to some degree, used to generate estimates from world organizations (41, 53, 60); iv) studies that focused on “less recent” data [that we defined as before 1995 – **Table 1** and (40, 42, 46, 50, 54, 55, 57, 62, 67)]. Among the remaining studies (n=15), only one found that under-reporting was minimal [in forty-seven Japanese prefectures (43)]. The remaining fourteen studies are reported in **Table 2**. For each country and time period in **Table 2**, we performed web-scraping, through references of the studies included in this review, WHO Suicide Worldwide documents (2, 79), Global Burden of Diseases datasets (80), to retrieve data on the mean number of suicides (or, alternatively mean population size and mean suicide rate). Data from the countries/regions reported in **Table 2** (Australia, Canada, China, Denmark, India, Israel, Kenya, Norway, South Africa, Sweden, Taiwan, UK, and USA), were used to run a random-effects meta-analysis (14 studies) estimating suicide underreporting (see also Supplementary Materials). The overall pooled underreporting rate was 15.8% (95% CI: 8.9–26.7%), with high heterogeneity (τ²=1.582, I²=99.99%, AIC=46.94) and a wide 95% prediction interval ranging from 1.4% to 70.7%.

**Table 2:** Number of suicides raw and corrected for underreporting for fourteen studies with non-zero suicide underreporting.

First author	Country/Region	Years considered	Mean n. suicides/year	Mean n. UR suicides/year	Mean n. suicides/year UR-corrected	Mean % of UR	CI lower	CI upper	NOS Score	WHO Data Quality Tier
Chang S.	Taiwan	1971-2007	2,111.5	905	3,016.5	30	28.1	32	6	4
Bakst	Israel	2005-2008	391.3	162.9	554.2	29.4	25.1	34.1	8	1
Li	China	2004-2016	147,683.6	34,192.8	181,876.4	18.8	18.6	19	2	4
Sampson	UK	1992-1997	3,715	987	4,702	21	19.7	22.3	3	1
Burrows	South Africa	2000-00	8,242.7	1,124	9,366.7	12	11.3	12.7	6	2
Bose	South India	1998-2004	64,800*	66,374*	131,174*	50.6*	50.2	50.9	5	4
Klugman	USA	1999-2002	29,400*	2,907.6*	32,307.6*	9*	8.7	9.3	3	1
Cox	USA	2005-2009	34,200 (81,82)	3,054.9	37,254.9	8.2	7.9	8.5	2	1
Tøllefsen	Norway, Sweden, Denmark	2008	2,698.7	203.1	2,901.8	7	6.1	8	8	1
Auger	Canada	1991-2001	4,332.7	481.5	4,814.2	10	9.1	10.9	4	1
Arya	India	2005-2015	125,975	72,971	198,946	37	36.7	37.3	8	4
Cantor	Australia	1990-1995	2,358	137.2	2,495.2	5.5	4.6	6.5	6	1
Gatov	Canada	2003-2012	3,680*	44.6*	3,724.6*	1.2*	0.9	1.6	5	1
Ongeri	Kenya	2011-2017	3,014	2,838.4	5,852.4	58.5	56.7	60.2	4	4
**Sum of suicides (or underreporting mean)**			432,602.5	186,384	618,986.5	21.5 ± 15.4%				

CI, Confidence Interval; NOS, Newcastle-Ottawa Scale; UR, underreported/underreporting; WHO, World Health Organization. Whenever possible, original data supplied by the authors, or references cited (81, 82), were used to compute the mean number of suicides per year in the period considered. In the other cases (Li et al., Burrows et al., Tøllefsen et al., Auger et al., Ongeri et al.), we integrated the data with the Global Burden of Disease estimates. Figures in bold represent data not used for estimation of overall underreporting due to either very low/very high rates or avoiding double representing a country (i.e., USA). The choice to exclude these studies was also support by the leave-one-out diagnostics reported in the **Supplementary Materials**. Figures in bold represent data not used for estimation of overall underreporting due toeither very low/very high rates or avoiding double representing a country (i.e., USA).

To explore potential sources of heterogeneity, a mixed-effects meta-regression was conducted using WHO data quality tier (high=1, medium=2, low=3 or very low=4) as a moderator. This stratified model explained approximately 45% of the between-study variance (R²=44.87%), reducing τ² to 0.872 and improving model fit (AIC=37.88). Underreporting estimates significantly differed across tiers (QM (2)=12.44, p=0.002), with Tier 4 countries (those with the lowest quality) displaying the highest average underreporting (β=1.87, 95% CI: 0.83–2.92, p<0.001), corresponding to a predicted underreporting rate of 37.9% (95% CI: 21.2–58.0%), compared to 8.6% (95% CI: 4.7–15.2%) in Tier 1 countries.

Newcastle-Ottawa Scale score (synthetizing original studies quality) was also tested as a continuous moderator in a separate meta-regression, but it did not significantly account for heterogeneity (QM (1)=0.097, p=0.76), and no reduction in τ² was observed (R²=0%). When inverse risk-of-bias weighting was applied (i.e., weighting studies proportionally to their NOS scores), the overall pooled underreporting estimate remained similar (16.4%, 95% CI: 8.8–28.6%), further supporting the robustness of the previous finding.

**Figure 3:**
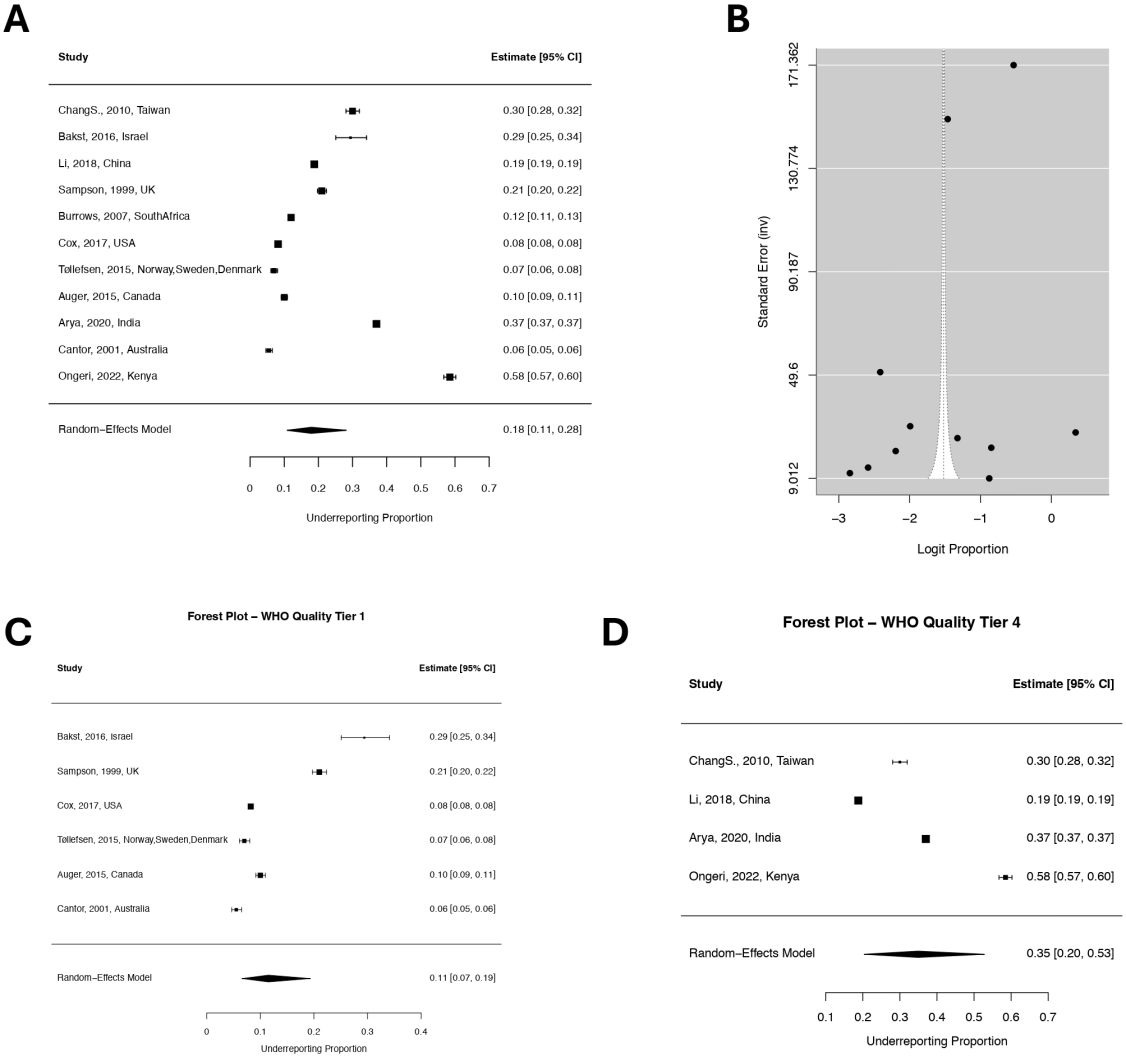
Forest and funnel plots stratified by WHO data quality tiers. **(A)** Forest plot of the estimated proportion of suicide underreporting with 95% confidence intervals among eleven studies. **(B)** Funnel plot of the logit-transformed underreporting proportions (x-axis) against the inverse standard error (y-axis), The asymmetric distribution of the studies around the funnel suggests the possibility of small-study effects or selective reporting. **(C)** Forest plot for studies assigned to WHO Quality Tier 1. **(C)** Forest plot for studies assigned to WHO Quality Tier 4.

A leave-one-out analysis identified two highly influential studies—Bose et al. (69), Gatov et al. (15)—with significant impact on τ² and Cook’s distance. A leave-one-out sensitivity analysis (**Figure 3**) excluding these two studies and the study by Klugman et al. [to avoid sampling US data twice (61)] (n=11) yielded a slightly increased pooled estimate of 17.9% (95% CI: 10.9–28.1%), with reduced heterogeneity (τ²=0.97, AIC=32.10). Stratified analyses in this reduced dataset again confirmed significantly higher underreporting in Tier 4 countries (34.9%, 95% CI: 20.3–53.0%) compared to Tier 1 (11.5%, 95% CI: 6.6–19.3%; QM (2)=8.74, p=0.013). South Africa was the only country in Tier 2 (estimated underreporting 12% 95% CI: 11–13%). No data from countries belonging to Tier 3 was available for meta-analysis.

The mean number of suicides for the pooled sample of country-period pairs was 432,602.5 Although this figure accounts for approximately 61.5% of suicides happened in 2019, and it yielded an estimated underreporting rate ranging from 10.9% and 28.1% (corresponding to roughly 86,000–275,000 suicides being underreported each year), it assumes that suicide rates (and thus under-reporting) are invariant in the same country/regions, which does not apply to countries like India (69, 70). In fact, if we considered that the mean underreporting rate for suicide in the Indian urban population would be 34.9% (for a total population of approximately 455 million people living in urban areas) and 50.6% would apply to the remaining 63.64% of the Indian population [the mean under-reporting rate in peri-urban/rural areas of India (69)], the number of underreported suicides in India alone would increase by approximately 55,000. To provide a more nuanced global estimate, we queried the 2021 WHO document (2) to retrieve information on the quality of suicide data for all other countries which were not assessed in this systematic review. Quality 2 and 3 (as per 2019 WHO document, referred to as quality “medium” and “low”, respectively, in the latest document), as per WHO definition, means that “*multiple years of death registration data are available. Data have low completeness and/or issues with cause-of-death assignment which likely affect estimated deaths by cause and time trends*” (n=50 countries), whereas Quality 4 (2021 label is “very low”) means that “*death registration data are unavailable or unusable due to quality issues*” (n=75 countries including China, India, Indonesia and Kenya). To compute an estimate of suicide underreporting for these countries (n=183), we used as a reference the number of suicides reported in 2021 published by the WHO this year (number of suicides=727,000). For countries belonging to quality tier 3 and 4, we applied a mean estimate suicide under-reporting of 34.9% (based on the under-reporting rate derived the meta-analysis) and both lower (20.3%) and upper (53%) confidence interval limits. For countries with data quality 1 and 2 we applied an estimate of 11.5% (and lower/upper confidence interval limits of 6.6 and 19.3%). Considering the lower limit of the confidence interval, the lowest number of underreported suicides to expect for 2021 in tier 3 and 4 countries (n=97) would be 112,703; the mean number of underreported suicides according to the meta-analytical mean would be 237,213; and the highest number would be almost half a million (498,967). On the other hand, considering the lower limit of the confidence interval, the lowest number of underreported suicides to expect for 2021 in tier 1 countries (n=86) would be 19,807; the mean number of underreported suicides according to the meta-analytical mean would be 36,424; and the highest number would be 67,038.

Thus, we found a mean excess of 273,638 (range 132,511–566,006) deaths by suicide (out of 722,794 suicide deaths available for the analysis from the WHO report). Therefore, under the assumption of spatio-temporal-invariance of suicide underreporting across countries belonging to the same WHO data quality tier, a more empirically grounded estimate of the yearly number of deaths by suicide would be closer to one million (specifically 1,000,638 deriving from 996,432 + 4,206 – the mean excess we calculated plus the difference between the 727,000 WHO figure and the 722,794 figure extracted from the 183 countries in the WHO document), ranging between 855,305 (+4,206) and 1,288,800 (+4,206).

## Discussion

The under-reporting of suicide has been a persistent challenge in epidemiology. Official statistics, while essential for national and international comparisons, are reported to misclassify suicides within other cause-of-death categories, including UnD, ill-defined causes, and accidental deaths (3–5). The implications of this under-reporting are far-reaching, as it diminishes the perceived magnitude of suicide as a public health issue and undermines efforts to allocate appropriate resources for prevention and intervention (16, 17). Sociocultural and legal factors compound this issue, with stigma, financial repercussions such as life insurance invalidation, and systemic limitations in death certification processes contributing to the under-reporting of suicides (18–21). Some authors (or countries) have adopted various strategies to address under-reporting, including combining suicides with UnD and improving medico-legal investigations (16, 72). However, challenges persist, particularly in categorizing deaths by an ambiguous manner (35–37). Despite progress in identifying and addressing factors contributing to suicide under-reporting, systematic evaluations of the reliability and validity of suicide data remain scarce. This systematic review and meta-analysis synthesizes evidence covering 71 countries over a 122-year period, which was reported by 42 studies published between 1978 and 2024. The studies predominantly utilized retrospective data collected by governments, organizations, or consortia, highlighting a reliance on existing institutional datasets. None of the studies employed a prospective study design.

The sample sizes varied widely, reflecting the scope and focus of individual studies: Some researchers focused on countries, while others had access to individual-level data on suicides.

Demographic variables such as age and sex were inconsistently reported. Most of the studies focused on populations aged 15 years or older (**Figure 2**), while specific age ranges (e.g., 17–73). were considered in others (42, 50). Gender distribution was often omitted, but peripartum suicide was uniquely explored in two studies (41, 53). The gender distribution across studies that reported this data ranged from 15% females (61) to 52.8% (49). The studies employed various methodologies to estimate suicide under-reporting. Common comparators included UnD, unallocated causes, accidental deaths, or databases and internal controls. UnD and unknown causes were the most frequently used comparators (25 studies). Accidental deaths were employed in 18 studies, while re-examination of suicide data or comparisons across databases were prevalent approaches (44, 53, 58, 60, 64). Despite the widespread reliance on the International Classification of Diseases (ICD) for standardization, 14 studies did not explicitly reference the use of ICD codes (20, 32, 37, 39, 42, 46, 47, 55, 61, 63, 65, 67, 69, 70). This omission could limit the comparability of findings, as consistent coding practices are crucial for a reliable classification of causes of death. Autopsy rates emerge as a critical determinant of reporting accuracy. In Europe, each 1% increase in autopsy rates is correlated with higher recorded suicide rates (51). This relationship underscores the necessity of forensic investigations in reducing misclassification. In Africa and Southeast Asia, where autopsy rates are minimal, reliance on verbal autopsies and alternative data sources, such as hospital records (41, 70–72), serves as an imperfect substitute. The variability in forensic resources globally highlights an area ripe for investment and capacity building.

### Geographic disparities in literature on suicide under-reporting

Five studies focused on aggregated territories to study the patterns of suicide underreporting and its potential moderators. For instance, Pritchard and collaborators (17) analyzed WHO Annual Mortality Statistics across 20 Western countries and observed strong correlations between suicide and UnD death rates, particularly in older adults. Their findings suggested that higher UnD death rates in predominantly Catholic countries could reflect the influence of religiosity on the misclassification of suicides. Moens et al. (50) examined WHO mortality rates in 21 European countries, focusing on adolescents and young adults. The study estimated suicide under-reporting rates between 13% (males) and 20% (females) for 1980–1984. However, these figures likely overestimate under-reporting, as the authors assumed that all UnD and accidental poisoning deaths were suicides. Tøllefsen et al. (48) analyzed suicide death registers from Norway, Sweden, and Denmark, pairing 1,800 deaths from various causes by method to control for potential biases in misclassification. Expert reclassification revealed significant divergence, particularly between psychiatrists and expert coders, concerning the categorization of suicides as UnD deaths. Instead of “manual” reclassification, Liu et al. (39) leveraged machine learning to reclassify UnD in Utah, estimating a suicide under-reporting rate of 33%. In Canada, Aldridge and St. John (42) and Malla and Hoenig (67) reported underreporting rates of 42.8% for adolescent deaths and 11.9-41.1% for unexplained deaths in Newfoundland and Labrador.

The studies conducted in Asian territories reveal significant variability in suicide under-reporting, influenced by regional practices and the quality of death certification systems. In Malaysia, Maniam demonstrated an under-reporting rate of 81.2% by correlating violent UnD with suicides, highlighting the impact of classification practices such as the introduction of a new version of the International Classification of Diseases, on suicide reporting (6). In Portugal, De Castro (40) noted a significant increase in “controversial” cases of death after the adoption of ICD-9 in 1979, which changed introduced new nosographic categories and altered pre-existing ones, suggesting a suicide under-reporting rate of 30%. This underscores the impact of changes in classification systems on recorded suicide rates.

In Japan, Matsubayashi and Ueda found no strong evidence for suicide under-reporting based on analyses of vital statistics across 47 prefectures (43). Their study suggested that UnD or unknown causes were not significantly correlated with suicide rates or socioeconomic indicators, indicating relatively robust classification practices in this context (43). However, this contrasts with findings from Taiwan, where Chang et al. estimated at least a 30% under-reporting rate between 1990 and 2007, with misclassification primarily occurring in categories such as accidental poisoning or suffocation (16).

Across all continents, the consistent under-representation of low- and middle-income countries (LMICs) is a notable gap. Western countries, especially in Europe and North America, dominate the literature, probably benefiting from more robust infrastructure for vital statistics and death certification. For example, studies from Norway (37) and Canada (15) demonstrate the availability of detailed data, allowing for nuanced analyses of under-reporting trends over time. In contrast, African nations such as Kenya (71) and Southeast Asian regions such as Indonesia (44) need to rely on verbal autopsies or police records to highlight mark inconsistencies in official sources of suicide data and data collected locally. This geographical imbalance highlights a critical gap in research on under-reporting in low- and middle-income countries, where healthcare infrastructure and death certification processes may differ significantly from those in high-income countries.

In general, a large amount of resources has been invested into studying the “hidden” suicide deaths in the UnD category, providing convincing evidence that, in countries with fewer medico-legal investigations, a significant proportion of UnD are in fact suicides.

### Sociocultural and gender disparities

Gender and age disparities are recurring themes. Studies from Poland (22) and Canada (56) show higher under-reporting rates among women, potentially due to societal assumptions about gender and suicide and the use of less violent means by females. Similarly, younger populations and maternal suicides are often not sufficiently addressed, as seen in Hong Kong (41) and the Netherlands (53). These findings parallel patterns in Africa, where younger individuals’ suicides are disproportionately misclassified (47, 71). Moreover, suicide rates for black adolescents in the US have been shown to be underestimated due to systemic under-reporting, narrowing the perceived disparity between black and white adolescent suicide rates when corrected (18, 32). Additionally, individuals without documented mental health conditions—often from minority groups—are more likely to have their deaths misclassified, underscoring the need for equitable practices in death certification (32, 59). Arya et al. provided a broader perspective on gender and suicide under-reporting by comparing Indian Vital Statistics with Global Burden of Disease (GBD) estimates, finding a 37% under-reporting rate, with significant gender and age disparities—female suicides were under-reported by up to 54%, and younger and older individuals were more likely to be misclassified (70). The enduring influence of social, cultural, and legal factors exacerbates the issue of under-reporting. Stigma, fear of invalidated life insurance claims, and other societal pressures often discourage accurate reporting of suicides (18). Similarly, systemic limitations, such as inadequate training of certifying officials, incomplete data collection processes, and cultural or religious biases, can lead to misclassification of deaths as non-suicides (10, 11). These influences are particularly evident in the variance of misclassification rates by the method of death; for example, suicides by hanging are more accurately reported than those involving less overt means, such as poisoning (16, 23). Religiosity also emerged as a recurring theme in suicide under-reporting. Pritchard et al. (52) extended their investigation to four Orthodox/Catholic European countries and 21 predominantly Islamic countries, using WHO mortality data. They found that 19 of 21 Islamic countries had UnD and accidental death to suicide rate ratios exceeding 2, with nearly half exceeding a ratio of 15. These findings strongly suggest substantial under-reporting in countries where religiosity or cultural norms may stigmatize suicide. Similarly, Catholic-majority countries such as Ireland and Poland exhibit significant under-reporting linked to religious doctrines that stigmatize suicide (29, 55). Interestingly, reported suicide rates in 16 of these countries were below 3 per 100,000 population, about one-third of the global average, highlighting a significant discrepancy potentially driven by sociocultural and religious factors.

### Estimate of suicide under-reporting worldwide

The findings of this study provide a conservative estimate of the global under-reporting of suicide, highlighting significant disparities in the quality of suicide reporting across countries and regions. Our analysis reveals two mean estimates of global underreporting: one for WHO data quality tier 1 (high quality) countries (11.5%; 95% CI: 6.6–19.3%) and one for tier 4 (very low data quality) countries (34.9% 95% CI: 20.3–53.0%) which corresponds to approximately 273,638 (range 132,511–566,006) additional suicides annually. When these under-reported deaths are included, the global estimate of yearly suicide deaths rises from 727,000 (2) to over 1 million.

The highest rates of under-reporting were observed in countries with low-quality or incomplete death registration systems (e.g., India, China, Kenya, and other Quality 4 countries per WHO classification), in line with previous studies that highlighted the challenges of cause-of-death misclassification and incomplete data in LMICs (39, 70).

In India, for example, the application of distinct under-reporting rates for urban and rural populations highlights how socio-demographic factors contribute to the variability in suicide data. Rural areas, which account for the majority of the Indian population, show particularly high under-reporting rates [up to 77.3% (69)], likely due to cultural stigma, limited infrastructure, and inconsistent data recording practices (72). Similarly, in other Quality 4 countries, the lack of reliable death registration systems undermines the accuracy of suicide statistics (20, 68).

While high-income countries (e.g., Australia, Canada, and Sweden) exhibit relatively lower rates of under-reporting (11.5%), the results suggest that accurate suicide statistics require ongoing efforts to maintain and improve effective surveillance infrastructures. This is consistent with prior studies showing that some suicides (e.g., with low lethality means) are more likely misclassified as accidental or UnD (58). In contrast, countries with weaker death registration systems or cultural barriers to reporting suicide exhibit underreporting rates exceeding 30%, as shown in **Table 2**. The analysis also emphasizes the need for greater attention to under-reporting in middle-income countries, which account for a significant proportion of global suicides. Notably, the systematic under-reporting in countries like India and China—together accounting for approximately 40% of global *reported* suicides—underscores the need for targeted improvements in data quality and reporting mechanisms (51).

### Limitations

This systematic review and meta-analysis has several limitations that may influence the interpretation of its findings.

First, the studies reviewed used diverse methodological approaches, ranging from correlational studies to database linkage, with varying reliance on different versions of the ICD codes (62, 65, 72). Moreover, the “gold standards” to which suicide data was compared comprised mainly official data (e.g., vital statistics) and trends in other manners of death: many studies estimated under-reporting by reclassifying UnD or accidental deaths as suicides (5, 52). While this approach provides valuable estimates, it risks over- or underestimating under-reporting depending on the assumptions, as illustrated by Moens et al. (50), who assumed all accidental poisonings were suicides.

Second, substantial heterogeneity and methodological variation across studies was evident. Only 14 out of 42 studies were suitable for inclusion in the meta-analysis, due to diverse study designs, target populations, comparators used, and methods of estimating suicide underreporting. For example, some studies relied on verbal autopsies (71), others on administrative data linkage (15, 60), expert reclassification (48), or machine learning algorithms (39). The operational definitions of underreporting also varied, ranging from assuming all UnD are suicides to more conservative estimations. Although stratified analyses by WHO data quality tier accounted for some of this variance, methodological differences remained a significant source of heterogeneity.

Furthermore, research is disproportionately focused on some geographical regions and timeframes: Western countries such as the United States, Canada, and Europe (15, 17, 28, 50) account for approximately 75% of the studies included in this review, although less than 25% of the suicides included in this review happened in those regions. South America, Africa, and parts of Asia remain the most underrepresented (16, 47, 71) regions. Additionally, demographic data, including age, gender, and socio-economic status, were inconsistently reported, limiting analyses of diverse populations (41, 53). The review spans studies conducted over a century (1900–2021), with significant variation in data collection periods (44, 46). Historical changes in societal attitudes, classification systems (e.g., shifts from ICD-8 to ICD-10), and healthcare practices may bias trends, complicating interpretations of whether changes reflect actual suicide rates or methodological differences. For example, De Castro (40) reported a sudden increase in “controversial cases” after the adoption of ICD-9 in Portugal, implying a shift in classification practices that could alter underreporting estimates. Similarly, improvements in forensic capacity or reduced stigma in more recent decades may have decreased underreporting in certain countries. The temporal breadth of the data thus complicates the assumption of constancy in underreporting rates over time, which may influence our meta-analytic estimates.

Lastly, the estimate of global under-reporting we computed, although relying on a meta-analytical background stands on some assumptions for which it is challenging to test validity. The analysis assumed invariant under-reporting rates within regions and countries over time, which may not reflect changes in suicide reporting in the world. For example, urbanization and increased awareness of mental health issues in India could potentially reduce under-reporting in urban areas over time (68, 69); moreover, the exclusion of studies focusing on subpopulations or specific databases may have led to an underestimation of under-reporting rates in certain contexts (32, 47). Additionally, the inference that the meta-analytical estimate of underreporting, which was based on some countries, would apply to other countries belonging to the same/similar WHO data quality tier may introduce biases, as the WHO estimates are based on modelled data rather than direct observations (79). In this scenario, we considered that countries with low data quality had similar underreporting rates of countries with very low data quality; on the other hand, we balanced this choice by considering the underreporting rate of medium data quality countries to be similar to that of high data quality countries. This decision probably led to underestimating the burden of suicide misclassification in countries with tier 2/medium quality.

## Conclusion

This study highlights that global suicide under-reporting ranges between 6.6% and 52% annually. Addressing under-reporting is crucial to assessing the true burden of suicide. This is particularly essential in low- and middle-income countries where most of suicide deaths occur. Improving the quality and consistency of suicide data should be a global priority, as it forms the foundation for effective suicide prevention and public health strategies worldwide. Suicide under-reporting is deeply intertwined with sociocultural, methodological, and systemic factors. While Western countries benefit from more consistent data and forensic resources, the gaps in LMICs heavily impact the reporting of suicide deaths, leading to suboptimal allocation of suicide prevention resources and failing to observe the efficacy of such interventions. Enforcing the use of international, shared coding (e.g., ICD) to reduce equivocality in the classification of equivocal deaths, addressing stigma, and improving forensic capacity can significantly enhance the accuracy of suicide statistics. By addressing these disparities, and further corroborating the finding that deaths by suicide each year worldwide are more likely to be approximately 1 million than 727,000, researchers and policymakers can better respond to the true magnitude of suicide, ultimately informing effective prevention strategies worldwide.

## Availability of data and materials

All data generated or analyzed during this study are included in this manuscript.

## Data Availability

The original contributions presented in the study are included in the article/supplementary material. Further inquiries can be directed to the corresponding author.
