# Supplementary material for: How many people die by suicide each year? Not 727,000: a systematic review and meta-analysis of suicide underreporting across 71 countries over 122 years
Source: Front Psychiatry. 2025 Aug 12;16:1609580. doi: 10.3389/fpsyt.2025.1609580 (PMC12378953; doi:10.3389/fpsyt.2025.1609580)
Supplement: Supplementary file 1 [file DataSheet1.docx]

**Supplementary Materials for:**

**How many people die by suicide each year? Not 727,000: a systematic review, and meta-analysis of suicide underreporting across 71 countries over 122 years**

**Authors:** Nicola Meda, MD ^1,2^, Ludovica Angelozzi MD ^1^, Matteo Poletto MD ^1^, Angelo Patane’ MD ^1^, Josephine Zammarrelli MSc ^2^, Irene Slongo MSc ^3^, Fabio Sambataro MD, PhD ^1,4,5^ Diego De Leo MD, PhD, DSc ^2,6,7,8^

**Affiliations:**

^1^ Department of Neuroscience, University of Padova, Padova, Italy

^2^ De Leo Fund, Padova, Italy

^3^ Independent researcher in Padova, Italy

^4^ Padova Neuroscience Center, University of Padova, Padova, Italy

^5^ Azienda Ospedale-Università Padova, Padova, Italy

^6^ Italian Psychogeriatric Association, Padova, Italy

^7^ Australian Institute for Suicide Research and Prevention, Griffith University, Brisbane, Australia

^8^ Slovene Centre for Suicide Research, Primorska University, Koper, Slovenia

Corresponding author:

Fabio Sambataro, MD, PhD

Department of Neuroscience (DNS), University of Padova

Via Giustiniani, 3, Padua, Italy

Tel: +390498211980

Email: [fabio.sambataro@unipd.it](mailto:fabio.sambataro@unipd.it)

ORCID: 0000-0003-2102-416X

**Index**

**PRISMA Checklist………………………………………………………………….………………3**

**PRISMA Abstract Checklist…………………………………………………………………….…3**

***Ad hoc* version of the Newcastle - Ottawa quality assessment Scale…………………………..…8**

**Table S1. ……………………………………………………………………………………...……11**

**Table S2. ………………………………………………………………………………………...…13**

**Table S3. ………………………………………………………………………………………...…15**

**Meta-analysis models, tables and figures………………………………………………………18**

**PRISMA** Checklist

| **Topic** | **No.** | **Item** | **Location where item is reported** |
| --- | --- | --- | --- |
| **TITLE** |  |  |  |
| **Title** | 1 | Identify the report as a systematic review. | 1 |
| **ABSTRACT** |  |  |  |
| **Abstract** | 2 | See the PRISMA 2020 for Abstracts checklist | 2 |
| **INTRODUCTION** |  |  |  |
| **Rationale** | 3 | Describe the rationale for the review in the context of existing knowledge. | 5 |
| **Objectives** | 4 | Provide an explicit statement of the objective(s) or question(s) the review addresses. | 5 |
| **METHODS** |  |  |  |
| **Eligibility criteria** | 5 | Specify the inclusion and exclusion criteria for the review and how studies were grouped for the syntheses. | 6 |
| **Information sources** | 6 | Specify all databases, registers, websites, organisations, reference lists and other sources searched or consulted to identify studies. Specify the date when each source was last searched or consulted. | 6 |
| **Search strategy** | 7 | Present the full search strategies for all databases, registers and websites, including any filters and limits used. | 6 |
| **Selection process** | 8 | Specify the methods used to decide whether a study met the inclusion criteria of the review, including how many reviewers screened each record and each report retrieved, whether they worked independently, and if applicable, details of automation tools used in the process. | 6 |
| **Data collection process** | 9 | Specify the methods used to collect data from reports, including how many reviewers collected data from each report, whether they worked independently, any processes for obtaining or confirming data from study investigators, and if applicable, details of automation tools used in the process. | 6 |
| **Data items** | 10a | List and define all outcomes for which data were sought. Specify whether all results that were compatible with each outcome domain in each study were sought (e.g. for all measures, time points, analyses), and if not, the methods used to decide which results to collect. | 6 |
|  | 10b | List and define all other variables for which data were sought (e.g. participant and intervention characteristics, funding sources). Describe any assumptions made about any missing or unclear information. | 6 |
| **Study risk of bias assessment** | 11 | Specify the methods used to assess risk of bias in the included studies, including details of the tool(s) used, how many reviewers assessed each study and whether they worked independently, and if applicable, details of automation tools used in the process. | 6,7 |
| **Effect measures** | 12 | Specify for each outcome the effect measure(s) (e.g. risk ratio, mean difference) used in the synthesis or presentation of results. | 7 |
| **Synthesis methods** | 13a | Describe the processes used to decide which studies were eligible for each synthesis (e.g. tabulating the study intervention characteristics and comparing against the planned groups for each synthesis (item 5)). | 6,7 |
|  | 13b | Describe any methods required to prepare the data for presentation or synthesis, such as handling of missing summary statistics, or data conversions. | 7 |
|  | 13c | Describe any methods used to tabulate or visually display results of individual studies and syntheses. | 7 |
|  | 13d | Describe any methods used to synthesize results and provide a rationale for the choice(s). If meta-analysis was performed, describe the model(s), method(s) to identify the presence and extent of statistical heterogeneity, and software package(s) used. | 7 |
|  | 13e | Describe any methods used to explore possible causes of heterogeneity among study results (e.g. subgroup analysis, meta-regression). | 7,8 |
|  | 13f | Describe any sensitivity analyses conducted to assess robustness of the synthesized results. | 7,8 |
| **Reporting bias assessment** | 14 | Describe any methods used to assess risk of bias due to missing results in a synthesis (arising from reporting biases). | 7,8 |
| **Certainty assessment** | 15 | Describe any methods used to assess certainty (or confidence) in the body of evidence for an outcome. | 7,8 |
| **RESULTS** |  |  |  |
| **Study selection** | 16a | Describe the results of the search and selection process, from the number of records identified in the search to the number of studies included in the review, ideally using a flow diagram. | 7, 8; Figure 1 |
|  | 16b | Cite studies that might appear to meet the inclusion criteria, but which were excluded, and explain why they were excluded. | Suppl. Material |
| **Study characteristics** | 17 | Cite each included study and present its characteristics. | 7-9, Table 1 |
| **Risk of bias in studies** | 18 | Present assessments of risk of bias for each included study. | Suppl. Material |
| **Results of individual studies** | 19 | For all outcomes, present, for each study: (a) summary statistics for each group (where appropriate) and (b) an effect estimate and its precision (e.g. confidence/credible interval), ideally using structured tables or plots. | 7-9, Table 1 |
| **Results of syntheses** | 20a | For each synthesis, briefly summarise the characteristics and risk of bias among contributing studies. | 10-24 |
|  | 20b | Present results of all statistical syntheses conducted. If meta-analysis was done, present for each the summary estimate and its precision (e.g. confidence/credible interval) and measures of statistical heterogeneity. If comparing groups, describe the direction of the effect. | 22-24 |
|  | 20c | Present results of all investigations of possible causes of heterogeneity among study results. | 24,25, Suppl. Material |
|  | 20d | Present results of all sensitivity analyses conducted to assess the robustness of the synthesized results. | 24,25, Suppl. Material |
| **Reporting biases** | 21 | Present assessments of risk of bias due to missing results (arising from reporting biases) for each synthesis assessed. | Suppl. Material |
| **Certainty of evidence** | 22 | Present assessments of certainty (or confidence) in the body of evidence for each outcome assessed. | 24,25, Suppl. Material |
| **DISCUSSION** |  |  |  |
| **Discussion** | 23a | Provide a general interpretation of the results in the context of other evidence. | 26 |
|  | 23b | Discuss any limitations of the evidence included in the review. | 32,33 |
|  | 23c | Discuss any limitations of the review processes used. | 32,33 |
|  | 23d | Discuss implications of the results for practice, policy, and future research. | 31-34 |
| **OTHER INFORMATION** |  |  |  |
| **Registration and protocol** | 24a | Provide registration information for the review, including register name and registration number, or state that the review was not registered. | 5 |
|  | 24b | Indicate where the review protocol can be accessed, or state that a protocol was not prepared. | 5 |
|  | 24c | Describe and explain any amendments to information provided at registration or in the protocol. | 7 |
| **Support** | 25 | Describe sources of financial or non-financial support for the review, and the role of the funders or sponsors in the review. | 33 |
| **Competing interests** | 26 | Declare any competing interests of review authors. | 33 |
| **Availability of data, code and other materials** | 27 | Report which of the following are publicly available and where they can be found: template data collection forms; data extracted from included studies; data used for all analyses; analytic code; any other materials used in the review. | 33 |

**PRISMA** Abstract Checklist.

| **Topic** | **No.** | **Item** | **Reported?** |
| --- | --- | --- | --- |
| **TITLE** |  |  |  |
| **Title** | 1 | Identify the report as a systematic review. | Yes |
| **BACKGROUND** |  |  |  |
| **Objectives** | 2 | Provide an explicit statement of the main objective(s) or question(s) the review addresses. | Yes |
| **METHODS** |  |  |  |
| **Eligibility criteria** | 3 | Specify the inclusion and exclusion criteria for the review. | No |
| **Information sources** | 4 | Specify the information sources (e.g. databases, registers) used to identify studies and the date when each was last searched. | No |
| **Risk of bias** | 5 | Specify the methods used to assess risk of bias in the included studies. | No |
| **Synthesis of results** | 6 | Specify the methods used to present and synthesize results. | No |
| **RESULTS** |  |  |  |
| **Included studies** | 7 | Give the total number of included studies and participants and summarise relevant characteristics of studies. | Yes |
| **Synthesis of results** | 8 | Present results for main outcomes, preferably indicating the number of included studies and participants for each. If meta-analysis was done, report the summary estimate and confidence/credible interval. If comparing groups, indicate the direction of the effect (i.e. which group is favoured). | Yes |
| **DISCUSSION** |  |  |  |
| **Limitations of evidence** | 9 | Provide a brief summary of the limitations of the evidence included in the review (e.g. study risk of bias, inconsistency and imprecision). | Yes |
| **Interpretation** | 10 | Provide a general interpretation of the results and important implications. | Yes |
| **OTHER** |  |  |  |
| **Funding** | 11 | Specify the primary source of funding for the review. | No |
| **Registration** | 12 | Provide the register name and registration number. | No |

***AD HOC* VERSION OF THE NEWCASTLE - OTTAWA QUALITY ASSESSMENT**

Each “**🟑**” represents a +1 point on a scale 0-10 for quality of evidence on underreporting.

**Selection**

1) Representativeness of the exposed cohort

a) Whole sample included (e.g., Vital Statistics, full coverage of target population) **🟑🟑**

b) Sample reasonably representative of the target population **🟑**

c) Convenience sample or other

2) Selection of the non exposed cohort

a) Whole sample included (e.g., Vital Statistics, full coverage of control population) **🟑🟑**

b) Sample reasonably representative of the control population **🟑**

c) Convenience sample or other

3) Quality of Controls/comparator for suicide under-reporting

a) Individual patient data linkage across databases **🟑🟑**

b) National Vital Statistics or Local or regional database of suicide statistics **🟑**

c) Unintentional/accidental deaths statistics

d) Assumption that all undetermined intent deaths are instead suicides

4) Type of study

a) Prospective **🟑**

b) Other

**Comparability**

1) Comparability of cohorts on the basis of the design or analysis

a) Under-reporting percentages or estimates could be computed from presented data **🟑**

b) Under-reporting estimates were inferred

2) Basic demographics (age, %gender) of the suicide and control cohorts were reported

a) Yes **🟑**

b) No

**Outcome**

1) Misclassification as the primary outcome

a) Yes **🟑**

b) No

| **First author, year** | **Countries covered** |
| --- | --- |
| Kapusta et al.,2011 | Armenia, Austria, Azerbaijan, Belarus, Bulgaria, Croatia, Cyprus, Czech Republic, Denmark, Estonia, Finland, Georgia, Hungary, Iceland, Ireland, Israel, Kazakhstan, Kyrgyzstan, Latvia, Lithuania, Macedonia, Malta, Moldova, Netherlands, Norway, Portugal, Romania, Russian Federation, Slovakia, Sweden, Switzerland, Tajikistan, United Kingdom, Ukraine, Uzbekistan |
| Cheung et al.,2023 | Hong Kong |
| Rhodes et al.,2012 | Canada |
| Chang et al.,2010 | Taiwan |
| Bakst et al.,2016 | Israel |
| Li et al.,2019 | China |
| Matsubayashi et al.,2022 | Japan |
| Pritchard et al.,2015 | Australia, Austria, Canada, Denmark, Finland, France, Germany, Greece, Ireland, Italy, Japan, Netherlands, New Zealand, Norway, Portugal, Spain, Sweden, Switzerland, United Kingdom, United States of America |
| Breen et al.,2018 | Norway |
| Ali et al.,2022 | United States of America |
| Sun et al.,2013 | China |
| Onie et al.,2024 | Indonesia |
| Rockett et al.,2006 | United States of America |
| Sampson et al.,1999 | United Kingdom |
| Connolly et al.,1995 | Ireland |
| Burrows et al.,2007 | South Africa |
| Riblet et al.,2019 | United States of America |
| Lommerse et al.,2024 | The Netherlands |
| Bose et al.,2006 | India |
| Klugman et al.,2013 | United States of America |
| Allebeck et al.,1986 | Sweden |
| de Castro et al.,1989 | Portugal |
| Phillips et al.,1993 | United States of America |
| Cox et al.,2017 | United States of America |
| Liu et al.,2020 | United States of America |
| Tøllefsen et al.,2015 | Norway, Sweden, Denmark |
| Auger et al.,2016 | Canada |
| Donaldson et al.,2006 | United States of America |
| Moens et al.,1988 | Austria, Belgium, Bulgaria, Czechoslovakia, Denmark, England & Wales, Finland, France, Germany, Greece, Hungary, Iceland, Ireland, Luxembourg, Netherlands, Northern Ireland, Norway, Portugal, Scotland, Sweden, Switzerland |
| Arya et al.,2021 | India |
| Cantor et al.,2001 | Australia |
| Hoffmire et al.,2020 | United States of America |
| Cooper et al.,1995 | United Kingdom |
| Skinner et al.,2016 | Canada |
| Gatov et al.,2018 | Canada |
| Malla et al.,1983 | Canada |
| Aldridge et al.,1991 | Canada |
| Maniam et al.,1995 | Malaysia |
| Höfer et al.,2012 | Poland |
| Ongeri et al.,2022 | Kenya |
| Pritchard et al.,2020 | Azerbaijan, Bahrain, Bosnia, Egypt, Greece, Iran, Iraq, Ireland, Italy, Jordan, Kazakhstan, Kuwait, Kyrgyzstan, Malaysia, Morocco, Oman, Portugal, Qatar, Saudi Arabia, Tajikistan, Tunisia, Turkey, Turkmenistan, United Arab Emirates, Uzbekistan |
| Brugha et al.,1978 | Ireland |

**Table S1**. Study-country correspondence.

| **First author, year** | **Selection** | **Comparability** | **Outcome** | **Global** | **Risk of bias** |
| --- | --- | --- | --- | --- | --- |
| Kapusta et al.,2011 | 5 | 1 | 1 | 7 | ↓ |
| Cheung et al.,2023 | 5 | 0 | 1 | 6 | – |
| Rhodes et al.,2012 | 3 | 2 | 1 | 6 | – |
| Chang et al.,2010 | 3 | 2 | 1 | 6 | – |
| Bakst et al.,2016 | 5 | 2 | 1 | 8 | ↓ |
| Li et al.,2019 | 0 | 1 | 1 | 2 | ↑ |
| Matsubayashi et al.,2022 | 5 | 1 | 1 | 7 | ↓ |
| Pritchard et al.,2015 | 5 | 1 | 1 | 7 | ↓ |
| Breen et al.,2018 | 2 | 1 | 0 | 3 | ↑ |
| Ali et al.,2022 | 5 | 1 | 1 | 7 | ↓ |
| Sun et al.,2013 | 5 | 0 | 0 | 5 | – |
| Onie et al.,2024 | 5 | 2 | 1 | 8 | ↓ |
| Rockett et al.,2006 | 4 | 1 | 1 | 6 | – |
| Sampson et al.,1999 | 1 | 1 | 1 | 3 | ↑ |
| Connolly et al.,1995 | 5 | 0 | 1 | 6 | – |
| Burrows et al.,2007 | 5 | 0 | 1 | 6 | – |
| Riblet et al.,2019 | 1 | 0 | 1 | 2 | ↑ |
| Lommerse et al.,2024 | 5 | 0 | 0 | 5 | – |
| Bose et al.,2006 | 4 | 1 | 0 | 5 | – |
| Klugman et al.,2013 | 1 | 1 | 1 | 3 | ↑ |
| Allebeck et al.,1986 | 1 | 2 | 0 | 3 | ↑ |
| de Castro et al.,1989 | 2 | 1 | 1 | 4 | ↑ |
| Phillips et al.,1993 | 5 | 2 | 1 | 8 | ↓ |
| Cox et al.,2017 | 0 | 1 | 1 | 2 | ↑ |
| Liu et al.,2020 | 3 | 1 | 1 | 5 | – |
| Tøllefsen et al.,2015 | 5 | 2 | 1 | 8 | ↓ |
| Auger et al.,2016 | 3 | 0 | 1 | 4 | ↑ |
| Donaldson et al.,2006 | 2 | 2 | 1 | 5 | – |
| Moens et al.,1988 | 5 | 1 | 0 | 6 | – |
| Arya et al.,2021 | 5 | 2 | 1 | 8 | ↓ |
| Cantor et al.,2001 | 3 | 2 | 1 | 6 | – |
| Hoffmire et al.,2020 | 3 | 2 | 1 | 6 | – |
| Cooper et al.,1995 | 1 | 2 | 1 | 4 | ↑ |
| Skinner et al.,2016 | 0 | 0 | 1 | 1 | ↑ |
| Gatov et al.,2018 | 3 | 1 | 1 | 5 | – |
| Malla et al.,1983 | 1 | 0 | 1 | 2 | ↑ |
| Aldridge et al.,1991 | 1 | 2 | 1 | 4 | ↑ |
| Maniam et al.,1995 | 5 | 1 | 1 | 7 | ↓ |
| Höfer et al.,2012 | 5 | 1 | 1 | 7 | ↓ |
| Ongeri et al.,2022 | 3 | 0 | 1 | 4 | ↑ |
| Pritchard et al.,2020 | 5 | 0 | 1 | 6 | – |
| Brugha et al.,1978 | 0 | 0 | 1 | 1 | ↑ |

**Table S2**. NOS scores for the included studies. Risk of bias legend: “↓”=low risk of bias; “–”=moderate risk of bias; “↑”=high risk of bias

| **First author, year** | **DOI** | **Reason for exclusion** |
| --- | --- | --- |
| Rockett et al.,2022 | 10.1001/jamanetworkopen.2021.46591 | No estimates provide or only assumptions |
| Rockett et al.,2016 | 10.1001/jamapsychiatry.2016.1870 | No estimates provide or only assumptions |
| Salibet al.,2005 | 10.1002/gps.1211 | No estimates provide or only assumptions |
| Liet al.,2020 | 10.1007/s00127-020-01856-2 | No estimates provide or only assumptions |
| González-Andradeet al.,2011 | 10.1007/s10389-010-0372-4 | No estimates provide or only assumptions |
| Snowdonet al.,2022 | 10.1007/s11469-022-00932-9 | No target |
| Gamberiniet al.,2021 | 10.1007/s11739-020-02554-2 | Review/Letter/Panel/Survey |
| Rockettet al.,1993 | 10.1016/0277-9536(93)90388-K | No estimates provide or only assumptions |
| Paimanet al.,2017 | 10.1016/j.ajp.2017.01.004 | No estimates provide or only assumptions |
| Rockettet al.,2021 | 10.1016/j.eclinm.2021.100741 | No estimates provide or only assumptions |
| Bertuccioet al.,2024 | 10.1016/j.eclinm.2024.102506 | No estimates provide or only assumptions |
| Pompiliet al.,2012 | 10.1016/j.forsciint.2012.04.012 | Review/Letter/Panel/Survey |
| Magalhãeset al.,2018 | 10.1016/j.forsciint.2018.03.040 | No estimates provide or only assumptions |
| Utyashevaet al.,2022 | 10.1016/j.ijlp.2022.101796 | Review/Letter/Panel/Survey |
| Zhonget al.,2016 | 10.1016/j.jad.2016.09.003 | No estimates provide or only assumptions |
| Mewet al.,2017 | 10.1016/j.jad.2017.05.002 | Review/Letter/Panel/Survey |
| Abbaset al.,2018 | 10.1016/j.jad.2017.12.037 | No estimates provide or only assumptions |
| Liuet al.,2021 | 10.1016/j.jad.2021.01.024 | No estimates provide or only assumptions |
| Gusmãoet al.,2021 | 10.1016/j.jad.2021.04.048 | No estimates provide or only assumptions |
| Qiaoet al.,2022 | 10.1016/j.jad.2022.04.056 | No estimates provide or only assumptions |
| Chenet al.,2023 | 10.1016/j.jad.2023.02.006 | No estimates provide or only assumptions |
| Lekeiet al.,2014 | 10.1016/j.neuro.2014.02.007 | No estimates provide or only assumptions |
| Vichiet al.,2010 | 10.1016/j.psychres.2008.12.008 | No estimates provide or only assumptions |
| Lennonet al.,2020 | 10.1016/j.psychres.2020.113066 | No estimates provide or only assumptions |
| Yeloet al.,2021 | 10.1016/j.trf.2021.06.015 | No estimates provide or only assumptions |
| Nordrumet al.,2000 | 10.1016/S0379-0738(00)00163-8 | No estimates provide or only assumptions |
| Jonassonet al.,1998 | 10.1016/S0379-0738(98)00123-6 | No estimates provide or only assumptions |
| Pillay-van Wyket al.,2016 | 10.1016/S2214-109X(16)30113-9 | No estimates provide or only assumptions |
| Pritchardet al.,2007 | 10.1017/S0033291706009159 | No estimates provide or only assumptions |
| Huberset al.,2018 | 10.1017/S2045796016001049 | No estimates provide or only assumptions |
| Jordanset al.,2018 | 10.1017/S2045796017000038 | No estimates provide or only assumptions |
| Khanet al.,1998 | 10.1027/0227-5910.19.4.172 | No estimates provide or only assumptions |
| Razaeianet al.,2008 | 10.1027/0227-5910.29.3.164 | No estimates provide or only assumptions |
| Hurtadoet al.,2024 | 10.1038/s41598-024-54159-w | No estimates provide or only assumptions |
| Youniset al.,2023 | 10.1080/13623699.2023.2170580 | Review/Letter/Panel/Survey |
| Salibet al.,2005 | 10.1080/13651500510018257 | No estimates provide or only assumptions |
| Cantoret al.,1997 | 10.1080/13811119708258251 | No estimates provide or only assumptions |
| Kelleheret al.,1997 | 10.1080/13811119708258252 | No estimates provide or only assumptions |
| Connollyet al.,1997 | 10.1080/13811119708258253 | No estimates provide or only assumptions |
| Snowdonet al.,2020 | 10.1080/17441692.2020.1801789 | Review/Letter/Panel/Survey |
| Aggarwalet al.,2015 | 10.1093/bmb/ldv018 | No estimates provide or only assumptions |
| Chishtiet al.,2003 | 10.1093/eurpub/13.2.108 | No estimates provide or only assumptions |
| Moenset al.,1985 | 10.1093/ije/14.2.272 | No estimates provide or only assumptions |
| Hessøet al.,1987 | 10.1111/j.1600-0447.1987.tb10798.x | Review/Letter/Panel/Survey |
| Wasserrnanet al.,1998 | 10.1111/j.1600-0447.1998.tb10763.x | No estimates provide or only assumptions |
| Klecket al.,1988 | 10.1111/j.1943-278X.1988.tb00158.x | Review/Letter/Panel/Survey |
| Applebyet al.,1991 | 10.1136/bmj.302.6769.137 | No estimates provide or only assumptions |
| Branaset al.,2015 | 10.1136/bmjopen-2014-005619 | No estimates provide or only assumptions |
| Armstronget al.,2019 | 10.1136/bmjopen-2019-030836 | No estimates provide or only assumptions |
| Lachaudet al.,2018 | 10.1136/injuryprev-2017-042376 | No estimates provide or only assumptions |
| Lyonset al.,2020 | 10.1136/injuryprev-2020-043865 | No estimates provide or only assumptions |
| Toraleset al.,2023 | 10.1177/00207640231169650 | No estimates provide or only assumptions |
| Imet al.,2018 | 10.1177/0030222816675262 | Review/Letter/Panel/Survey |
| Snowdonet al.,2022 | 10.1177/10398562221075192 | No target |
| Mercadoet al.,2021 | 10.1177/19253621211022464 | Review/Letter/Panel/Survey |
| Krauset al.,2005 | 10.1179/107735205800245984 | No target |
| Rockettet al.,2010 | 10.1186/1471-244X-10-35 | No estimates provide or only assumptions |
| Tøllefsenet al.,2012 | 10.1186/1471-244X-12-9 | Review/Letter/Panel/Survey |
| Rockettet al.,2010 | 10.1186/1471-2458-10-705 | No estimates provide or only assumptions |
| Redanielet al.,2011 | 10.1186/1471-2458-11-536 | No estimates provide or only assumptions |
| Björkenstamet al.,2014 | 10.1186/1478-7954-12-11 | No estimates provide or only assumptions |
| Lewet al.,2022 | 10.1186/s12889-022-13101-3 | No estimates provide or only assumptions |
| Seabourneet al.,2001 | 10.1192/bjp.178.1.42 | No estimates provide or only assumptions |
| Rockettet al.,2015 | 10.1371/journal.pone.0135296 | No estimates provide or only assumptions |
| Rockettet al.,2018 | 10.1371/journal.pone.0190200 | No estimates provide or only assumptions |
| Jonassonet al.,1999 | 10.1520/jfs14458j | No estimates provide or only assumptions |
| Marín-Leónet al.,2012 | 10.1590/S1020-49892012001100005 | No estimates provide or only assumptions |
| Mohleret al.,2001 | 10.2105/AJPH.91.1.150 | No estimates provide or only assumptions |
| Carolloet al.,1996 | 10.2190/TE9C-VU3Q-TX0F-G471 | No estimates provide or only assumptions |
| Pageet al.,2010 | 10.3109/00048670903489874 | No estimates provide or only assumptions |
| Somanet al.,2009 | 10.4103/0019-5545.58290 | No estimates provide or only assumptions |
| Moradinazaret al.,2017 | 10.4178/epih.e2017023 | No estimates provide or only assumptions |
| Shahet al.,2009 | 10.5249/jivr.v1i1.40 | No estimates provide or only assumptions |
| De Leoet al.,2010 | 10.5694/j.1326-5377.2010.tb03584.x | No estimates provide or only assumptions |
| Tsaiet al.,2014 | 10.7326/M13-1291 | No estimates provide or only assumptions |
| Menghiniet al.,2000 | NAR | No estimates provide or only assumptions |
| Sauvagetet al.,2009 | NAR | No estimates provide or only assumptions |
| Bjorkenstamet al.,NAR | 10.1186/1478-7954-12-11 | No estimates provide or only assumptions |
| Gibsonet al.,NAR | NAR | No estimates provide or only assumptions |
| Sharmaet al.,NAR | NAR | Review/Letter/Panel/Survey |
| Kamineret al.,NAR | 10.1080/1067828X.2020.1837322 | No estimates provide or only assumptions |
| Frizeet al.,NAR | 10.1007/978-3-319-32703-7_159 | No estimates provide or only assumptions |
| Copleyet al.,NAR | NAR | No estimates provide or only assumptions |
| Estévezet al.,2024 | 10.1016/j.rcp.2023.12.005 | Language (Spanish) |
| Barberíaet al.,2018 | 10.1016/j.rpsm.2016.05.004 | Language (Spanish) |
| Coakleyet al.,1996 | 10.1017/S0790966700002652 | No estimates provide or only assumptions |
| Cullenet al.,1997 | 10.1080/13811119708258255 | No estimates provide or only assumptions |
| Minelliet al.,2013 | 10.1111/1556-4029.12114 | No target |
| Esscheret al.,2013 | 10.1111/aogs.12037 | No estimates provide or only assumptions |
| Bohnertet al.,2013 | 10.1136/injuryprev-2012-040631 | No estimates provide or only assumptions |
| Chaet al.,2016 | 10.1136/injuryprev-2015-041781 | No estimates provide or only assumptions |
| Donaldsonet al.,2006 | 10.1136/ip.2005.011171 | No estimates provide or only assumptions |

**Table S3.** DOI=Digital Object Identifier; NAR=Data Not Available when Retrieved; Studies excluded at the full-text screening level, and reason for exclusion.

# **Meta-Analysis Models**

## Overall Random-Effects (fourteen studies)

| Tau^2 | 1.5823 (SE = 0.6223) |
| --- | --- |
| Tau | 1.2579 |
| I^2 | 99.99% |
| AIC | 46.94 |
| Prediction Interval | 1.4% to 70.7% |

## Stratified by WHO Quality

| Tau^2 | 0.8723 (SE = 0.3739) |
| --- | --- |
| Tau | 0.9340 |
| I^2 | 99.97% |
| R^2 | 44.87% |
| AIC | 37.88 |
| QM | 12.44 (df = 2), p = 0.002 |

## NOS Score Meta-Regression

| Tau^2 | 1.5854 (SE = 0.6229) |
| --- | --- |
| Tau | 1.2580 |
| I^2 | 99.99% |
| R^2 | 0% |
| QM | 0.0972 (df = 1), p = 0.7554 |

## Inverse Risk Weighting (NOS-based)

| Pooled Estimate | 16.4% |
| --- | --- |
| 95% CI | 8.8% to 28.6% |
| Tau^2 | 1.6822 |
| I^2 | 99.99% |
| AIC | 49.23 |

**Figure S1. Forest plot of the meta-analytical estimate of suicide underreporting with 14 studies**

**Figure S2. Funnel plot corresponding to Figure S1 data**

**Figure S3. Leave-one-out analysis influence plot**. Number 8 and number 12 represent the studies by Gatov et al., and Bose et al., respectively and highlight that they either influenced significantly the predicted value of meta-analysis (Gatov et al.) or drove heterogeneity (Bose et al., and Gatov et al.).

# **Random-effects meta-analysis on the reduced dataset (eleven studies)**

## Leave-One-Out (Excl. 3 studies – Gatov et al., Bose et al., Klugman et al.)

| Pooled Estimate | 17.9% |
| --- | --- |
| 95% CI | 10.9% to 28.1% |
| Tau^2 | 0.97 |
| I^2 | 99.99% |
| AIC | 32.10 |
| QM (WHO) | 8.74 (df = 2), p = 0.013 |

## WHO Quality (Reduced Dataset)

| Tau^2 | 0.6614 (SE = 0.3268) |
| --- | --- |
| Tau | 0.8133 |
| I^2 | 99.94% |
| R^2 | 36.00% |
| AIC | 24.52 |
| QM | 8.74 (df = 2), p = 0.013 |
| Tier 4 Estimate | 34.9% (95% CI: 20.3–53.0%) |
| Tier 1 Estimate | 11.5% (95% CI: 6.6–19.3%) |

## NOS Score Meta-Regression (Reduced Dataset)

| Tau^2 | 0.9956 (SE = 0.4577) |
| --- | --- |
| Tau | 0.9978 |
| I^2 | 99.95% |
| R^2 | 0% |
| AIC | 32.27 |
| QM | 0.0814 (df = 1), p = 0.7753 |

## Inverse Risk Weighting (Reduced Dataset)

| Pooled Estimate | 16.5% |
| --- | --- |
| 95% CI | 9.3% to 27.5% |
| Tau^2 | 1.0628 |
| I^2 | 99.96% |
| AIC | 36.10 |
